# Influenza A virus vaccine research conducted in swine from 1990 to May 2018: A scoping review

**DOI:** 10.1371/journal.pone.0236062

**Published:** 2020-07-16

**Authors:** Sheila Keay, Zvonimir Poljak, Mackenzie Klapwyk, Annette O’Connor, Robert M. Friendship, Terri L. O’Sullivan, Jan M. Sargeant

**Affiliations:** 1 Department of Population Medicine, Ontario Veterinary College, University of Guelph, Guelph, Ontario, Canada; 2 Veterinary Diagnostic and Production Animal Medicine, College of Veterinary Medicine, Iowa State University, Ames, Iowa, United States of America; 3 Centre for Public Health and Zoonoses, Ontario Veterinary College, University of Guelph, Guelph, Ontario, Canada; Faculty of Science, Ain Shams University (ASU), EGYPT

## Abstract

**Background:**

Influenza A viruses of swine (IAV-S) are a global zoonotic and economic concern. Primary control is through vaccination yet a formal evidence map summarizing vaccine research conducted in pigs is not available.

**Objective:**

Ten characteristics of English language primary IAV-S vaccine research, conducted at the level of the pig or higher, were charted to identify research gaps, topics for systematic review, and coverage across different publication types.

**Design:**

Six online databases and grey literature were searched, without geographic, population, or study type restrictions, and abstracts screened independently and in duplicate for relevant research published between 1990 and May 2018. Full text data was charted by a single reviewer.

**Results:**

Over 11,000 unique citations were screened, identifying 376 for charting, including 175 proceedings from 60 conferences, and 170 journal articles from 51 journals. Reported outcomes were heterogeneous with measures of immunity (86%, n = 323) and virus detection (65%, n = 246) reported far more than production metrics (9%, n = 32). Study of transmissibility under conditions of natural exposure (n = 7), use of mathematical modelling (n = 11), and autogenous vaccine research reported in journals (n = 7), was limited.

**Conclusions:**

Most research used challenge trials (n = 219) and may have poor field relevance or suitability for systematic review if the purpose is to inform clinical decisions. Literature on vaccinated breeding herds (n = 89) and weaned pigs (n = 136) is potentially sufficient for systematic review. Research under field conditions is limited, disproportionately reported in conference proceedings versus journal articles, and may be insufficient to support systematic review.

## Introduction

### Rationale

Influenza A viruses in swine (IAV-S) are genetically and antigenically complex [1–3], globally enzootic [4], and are zoonotic pathogens [5]. Control of infection in swine is therefore a priority for both swine veterinarians [6–8] and human health researchers [9, 10]. Sustained elimination of influenza from swine populations is highly improbable [11] and vaccination has been a primary control intervention [2, 12–17]. IAV strains used in swine vaccines, and use of vaccines in swine populations is not regulated at a policy level as it is for certain subtypes in poultry populations, rather, the choice to vaccinate, and of which vaccine or combination of vaccines to use (i.e. prime-boost [18]), is largely producer controlled and guided by individual herd veterinarians [15, 19, 20]. As such the burden of keeping current with IAV-S vaccine research is borne by practicing veterinarians. Multiple IAV-S vaccines are licenced for use in swine, including autogenous vaccines (custom produced to match farm viral isolates), but messaging on vaccination programs vary and lacks consensus with respect to differing vaccine, target population, and herd management characteristics [15, 21, 22].

Given the importance and complexity of IAV-S, knowing how much and what kind of vaccine research evidence is a priority for professionals. Challenges for professionals in the field with staying current have been documented elsewhere [23–26]. Although comprehensive narrative reviews are available [7, 15, 27–30], the body of primary IAV-S vaccine research evidence has not been formally mapped using explicit and transparent search and charting methods [31].

### Objectives

Therefore, using scoping review methods, our objective was to chart and to summarize 10 characteristics from all English language primary IAV-S vaccine research conducted in swine at the level of the pig (*i*.*e*. individual animal, groups, or other levels of swine populations), and published between 1990 and May 22, 2018. Because we were also interested in what information was available to end-users based on the information sources they access, our objective was also to determine if reported categories differ by information source (peer reviewed journals versus conference proceedings). Secondary objectives were to identify research gaps, and potential questions for systematic review.

## Methods

### Protocol and registration

The protocol for this review titled ‘Protocol for a scoping review of Influenza A viruses infecting swine or directly related to swine’ was formatted as per the items in the PRISMA-P 2015 Checklist [32] and posted May 14, 2018, in advance of study commencement, on the University of Guelph Atrium https://atrium.lib.uoguelph.ca/xmlui/bitstream/handle/10214/13044/Keay_etal_2018_ProtocolScopingReviewIAV_SInSwine.pdf?sequence=3&isAllowed=y and on the website Systematic Reviews for Animals & Food (SYREAF) 2018 Protocols http://www.syreaf.org/contact/.

Initially, the stated objective was to chart a broader scope of IAV-S research, however, the protocol stated that if the search yielded too many citations the scope would be narrowed using additional relevance screening questions. The search output exceeded 18,000 citations and therefore the following protocol deviations were made:

1. Citations requiring manual entry of metadata were excluded.
2. After screening approximately 4000 citations, the scope was narrowed to only research conducted in live pigs.
3. Two questions were added at a second level of screening to narrow the scope to vaccine research only. This revised focus necessitated amendment of items for charting from the protocol; three additional charting items were added based on suitability as key elements in the formation of systematic review questions [33]; vaccine type, animal production stage at vaccination, and if the study reported population comorbidity.
4. Data were charted by a single reviewer.
5. Identification of proceedings published subsequently as journal articles was not performed.

### Revised eligibility criteria

English language full text publications of primary research from any geographic location were included without restrictions on study design. Publications dated prior to 1990 were excluded due to substantive changes that occurred since that time in diagnostics, swine production practices, and in the dynamic of swine diseases [34].

#### Population eligible/excluded

Research conducted at the level of the live pig or at the level of swine populations, either *in vivo* or *in silico* (i.e. mathematical modelling of vaccine interventions in swine populations), was included.

#### Intervention eligible/excluded

IAV-S vaccine research, inclusive of all vaccine types, regardless of the virus exposure of the vaccinated population (natural, purposeful challenge, or deliberate absence of exposure) was included.

#### Outcomes/comparators eligible/excluded

There were no restrictions on outcomes measured.

### Information sources

On May 21–22, 2018, five bibliographic databases were searched through four bibliometric platforms (Table 1). Swine veterinary association and collaboration websites, international collaborative reports, and swine conference proceedings and abstracts were hand searched (Table 2). The American Association of Swine Veterinarians (AASV) Online Swine Information Library [35] was searched May 25, 2018.

**Table 1:** Bibliographic databases and vendor interfaces (platforms) searched.

Platform	Database
CAB Direct	CAB Abstracts and Global Health-1973-current and others
PubMed	MEDLINE
Web of Science	The Science Publication Index, Clarivate Analytics, 1864-current-multiple databases
ProQuest	Agricola (USDA National Agricultural Library1970-Current)
ProQuest	Dissertations & Theses A&I: Health & Medicine Full Text (1998–2018)

**Table 2:** Grey literature sources searched.

**Swine veterinary associations and collaboration websites:**
i. The European Surveillance Network for Influenza in Pigs (ESNIP1,2and 3) projects reported on CORDIS (European Commission Community Research and Development Information Service) https://cordis.europa.eu, http://www.esnip.ugent.be/, https://www.wur.nl
ii. OFFLU–the joint OIE-FAO Network of expertise on animal influenza http://www.offlu.net
iii. STAR-IDAZ- the Strategic Alliances for the Cooperation of Research on the Major Infectious Diseases of Animals and Zoonoses http://www.star-idaz.net/
iv. The European Association of Porcine Health Management http://www.eaphm.org/
v. The American Association of Swine Practitioners (AASV) website https://www.aasv.org/
vi. The Swine Disease Eradication Center–The University of Minnesota https://www.vetmed.umn.edu/centers-programs/swine-program/research/industry-advisory-board
vii. The Swine Health Information Center https://www.swinehealth.org/
**International Collaborative Reports:** (references hand checked for inclusiveness of electronic bibliographic search output)
i. USDA**—**Animal Influenza Viruses Gap Analysis 2014 Animal Influenza Countermeasures Working Group (AICWG) workshop report
ii. EFSA—Workshop on Research Gap Analysis in Animal Influenza, January 2015 –event Report
iii. OFFLU and STAR-IDAZ–A consultation to Develop a Global Animal Influenza Research Agenda, Paris 2014
iv. Baudon et al. 2017. “Epidemiological features of influenza circulation in swine populations: A systematic review and meta-analysis.”
v. Freidl, G. S. et al. 2014. “Influenza at the Animal-Human Interface: A Review of the Literature for Virological Evidence of Human Infection with Swine or Avian Influenza Viruses Other than A (H5N1).”
**Conference Proceedings/Abstracts**
i. The International Society for Influenza and other Respiratory Virus Diseases (ISIRV) –https://isirv.org/site/
ii. American Association of Swine Veterinarians (AASV) Swine Information Library—A searchable digital catalogue available to members on the Association website of the following swine conference proceedings: http://www.aasv.org/library/swineinfo/
• AASV Annual Meeting: 1999–2018 • AASV Pre-Conference Seminars: 2007–2018 • International Pig Veterinary Society Congress (IPVS):2000, 2002, 2004, 2006, 2008, 2010, 2012, 2014, 2016 • Allen D. Leman Swine Conference: 1998–2017 • George A. Young Swine Health and Management Conference: 1999–2012 • International Symposium on Swine Disease Eradication: 2001–2002,2004 • ISU Swine Disease Conference for Swine Practitioners: 1999–2017

### Search

The search string formatted for Web of Science was as follows:

TS = (pork OR swine OR Sus scrofa OR pig$ OR piglet$ OR piglets OR gilt$ OR boar$ OR sow$ OR hog$ OR weaner$ OR feeder$ OR finisher$ OR “market-weight” OR porcine NOT "guinea pig$")
AND
TS = (“influenza*” OR “IAV” OR “Influenza A virus$” OR “swine influenza” OR “swine flu” OR “swine influenza virus” OR “SIV” OR “H3N2” OR “H1N1” OR “H1N2” OR “H3N1” OR “H2N3”)
(See also S1 Table)
The AASV Swine Information Library was searched using the key word ‘influenza’.

### Selection of sources of evidence (relevance screening), and data charting process

The search output was compiled and deduplicated using EndNote citation management software (© 2018 Clarivate Analytics) [36] and Microsoft Excel (2013) then uploaded to Distiller-SR (© 2018 Systematic Review and Literature Review Software by Evidence Partners). Three sequential levels of relevance screening (L1, L2, and L3) and data charting were conducted in Distiller using purpose built forms. Titles/abstracts were screened at L1 and L2 by two reviewers working independently. At L3, full text was screened by a single reviewer. Questions used in L1, and L2, and L3 relevance screening are detailed in supporting materials S2, S3 and S4 Tables, respectively. Notes of explanation for reviewers (S1 Text) and definitions (S5 Table) are detailed in supporting materials.

#### Data charting process and data items

Data on the following 10 characteristics were extracted:

1. Concurrent implementation of non-vaccine IAV-S control intervention(s).
2. Study design(s) employed.
3. Comorbidity of the study population.
4. Method(s) of exposure of study population to virus.
5. Vaccine type(s).
6. Production stage(s) of vaccinated study population(s).
7. Outcome(s) measured.
8. Primary author employment/research affiliation(s).
9. Primary author region/country affiliation.
10. Source(s) of research funding.

Charting questions and categorical response options are detailed in S4 Table. Category definitions are detailed in S5 Table. All applicable responses were charted for each publication (i.e. multiple responses per question were possible). Data were charted by a single reviewer. An additional team member with content expertise was consulted as needed.

### Synthesis of results

Data were downloaded from Distiller into Stata (StataCorp. 2015. Stata Statistical Software: Release 14. College Station, TX: StataCorp LP) for cleaning, analysis, and generation of summary frequency tables and bar graphs showing category publication counts by publication type. Data sub-sets were downloaded to Microsoft Excel (2013) for generation of a bubble plot and heat map charts. This information was used to identify candidate topics for potential systematic reviews. Systematic review intervention questions are typically a composite of four elements; population, intervention, comparator, and outcome [37].

## Results

### Selection of sources of evidence

**Fig 1:**
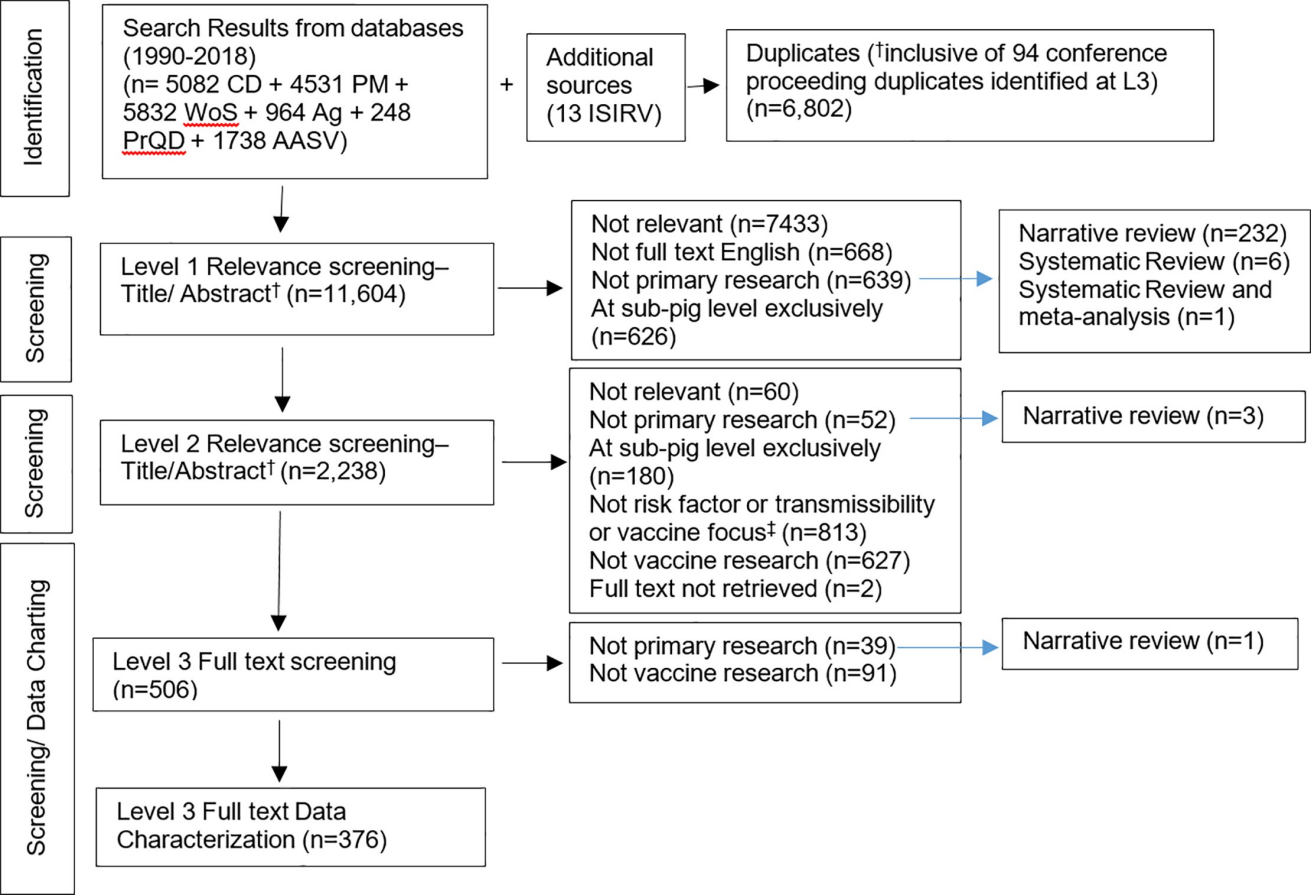
PRISMA flowchart describing the flow of literature through the review process. PRISMA = Preferred Reporting Items for Systematic Reviews and Meta-Analyses; CD = CAB Direct; PM = PubMed; WoS = Web of Science; Ag = Agricola; PrQD = ProQuest Dissertations; AASV = AASV Swine Information Library; ISIRV = IIRV conference events;^†^ AASV Swine Information Library publications title only screening at Level 1 and Level 2, duplicate publications identified at Level 3 full text screening; ^‡^ Publications were screened for content considered applicable to the field inclusive of risk factors for IAV-S infection, interventions to manage or control IAV including vaccines, or studies to estimate transmissibility parameters (See also S5 Table, S1 Appendix).

Results of the search and screening are summarized in the PRISMA flow diagram (Fig 1). The search strategy produced 11,604 unique citations, 7,493 were excluded as not relevant, for an overall screening yield of 376 publications. Proportionately higher exclusions at level 3 (130/506, 26%) was a function of screening AASV swine proceedings by title only at levels 1 and 2. Two dissertations could not be retrieved.

### Results of individual resources

Journals articles were published across 51 different academic journals (See S1 Fig). Forty-five percent (n = 76) were from 5 journals (Vaccine (n = 36), Veterinary Microbiology (n = 14), PLoS ONE (n = 11), Veterinary Immunology and Immunopathology (n = 8) and Journal of Virology (n = 7)), and 61% (n = 103) were published within 10 journals. The Directory of Open Access Journals (DOAJ) https://doaj.org/ was searched for each of the 51 journal titles. We found 10 which were listed on DOAJ, which cumulatively included18% of the journal articles (n = 31) (see S6 Table). The Journal of Swine Health and Production, although not listed on DOAJ, is also open access.

Relevant conference proceedings were from 14 different conference series spanning 60 events (see S7 Table). Most were from the AASV Annual Meeting (75, 43%), the IPVS (48, 27%) and the Allen D. Leman Conferences (25, 16%). Less than a third (50/175, 29%) were indexed by traditional publication portals and capture for indexing was irregular, missing proceedings from consecutive events in conference series. Almost 3/4 of relevant proceedings were sourced from the AASV Swine Information Library (125, 71%) and accessed through membership only.

Neglected Influenza Viruses Group (NIVG) conference proceedings were largely inaccessible and were not indexed by bibliographic databases with the exception of publication of selected conference full paper presentations from 2010 and 2013 events in the isirv Journal. Proceedings from 2013 and 2018 events, and the 2015 conference program were available only online to isirv members, and were accessed through membership. Thirty-five potentially relevant oral swine presentations (proceedings for 2013 (n = 19) and 2018 (n = 18), and titles only for 2015 (n = 16)) were omitted from further consideration in this the review due to lacking digitally accessible meta-data.

### Synthesis of results

**Fig 2:**
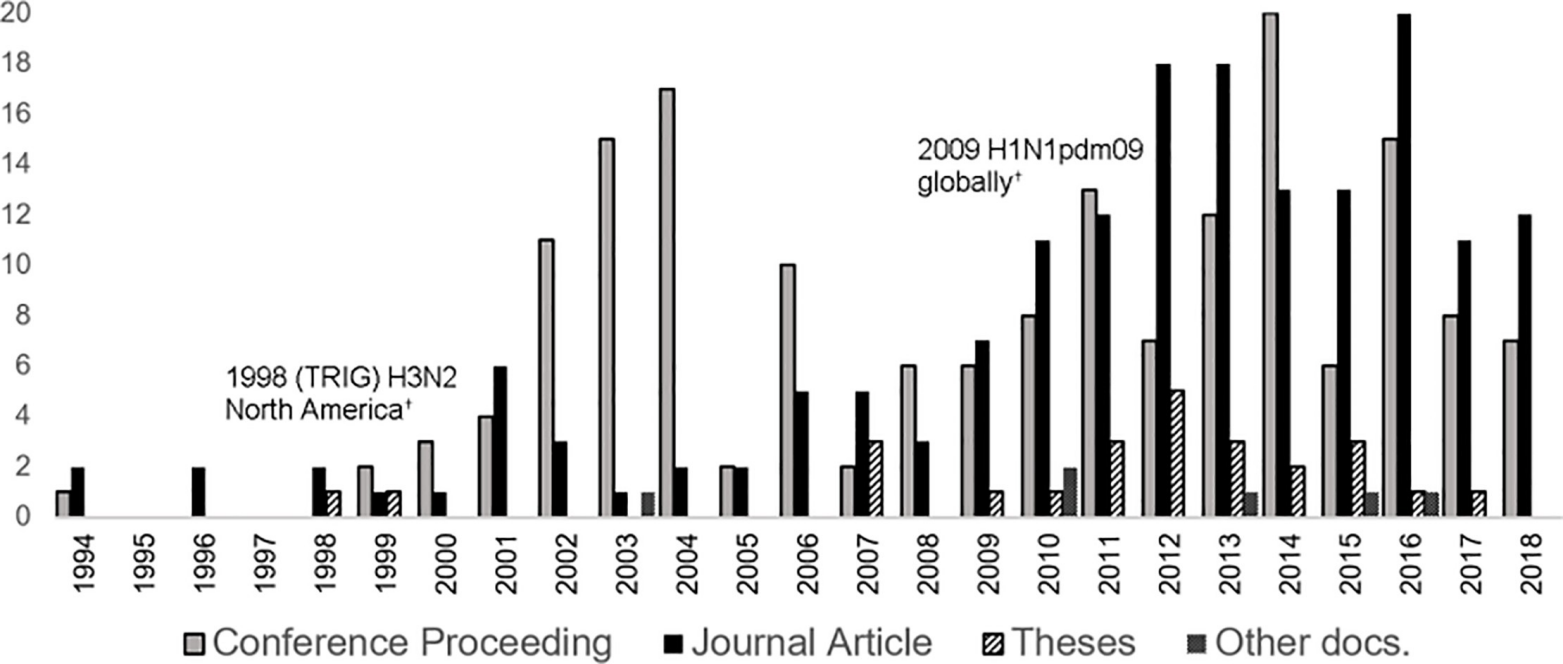
Counts of IAV-S vaccine research in pigs by publication type and year. Conference proceedings (N = 175); Journal articles (N = 170); Theses (N = 25): Other documents (N = 6). ^†^From: Vincent et al. 2014. Zoonoses and Public Health.**61**.1.pg4-17.

**Fig 3:**
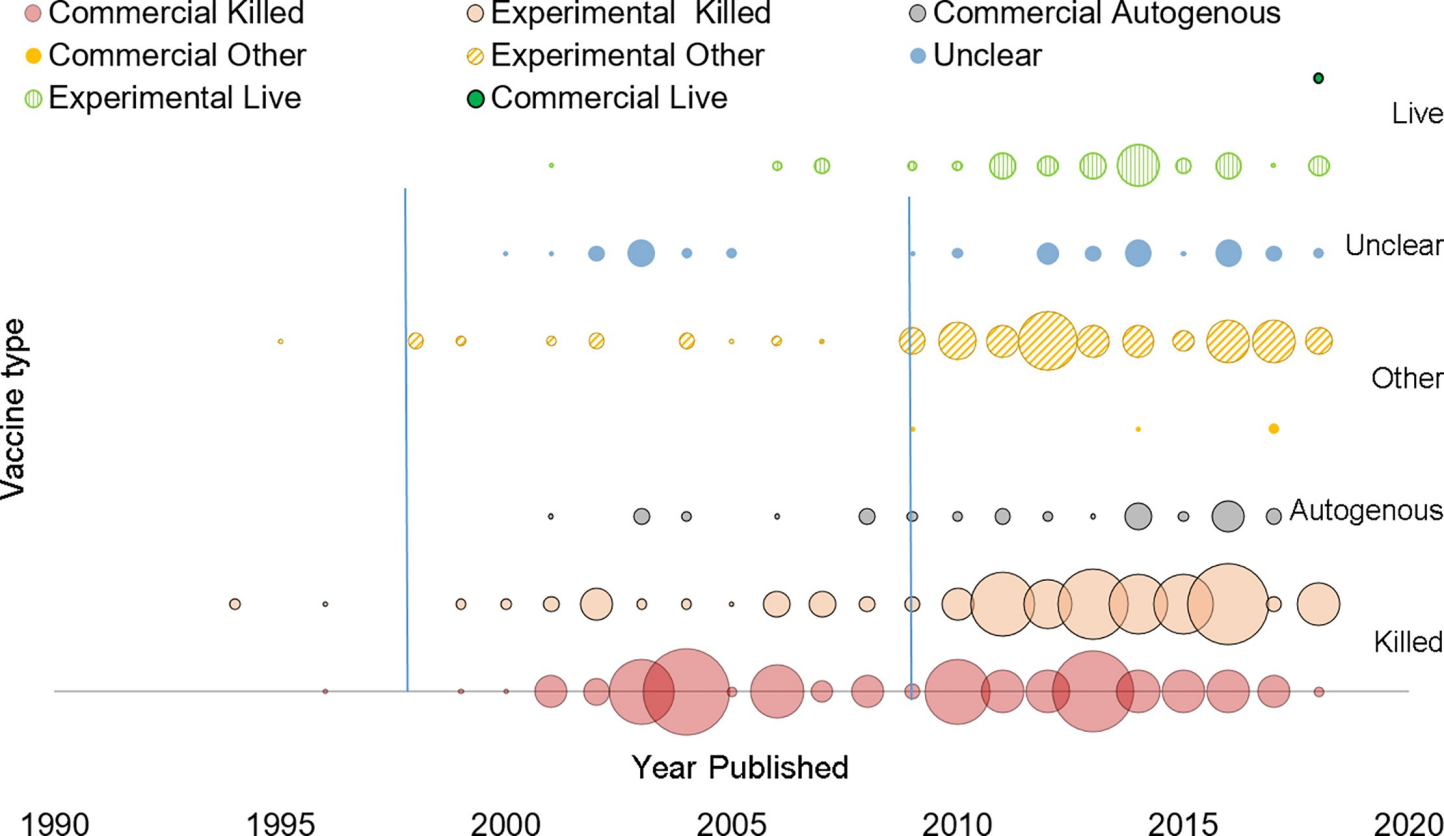
Bubble plot of IAV-S vaccine research publications by reported vaccine type and publication year. The size of the bubble is proportional to the number of publications published per year reporting on that vaccine type (See S5 Table, Table 2 for supporting data).

**Fig 4:**
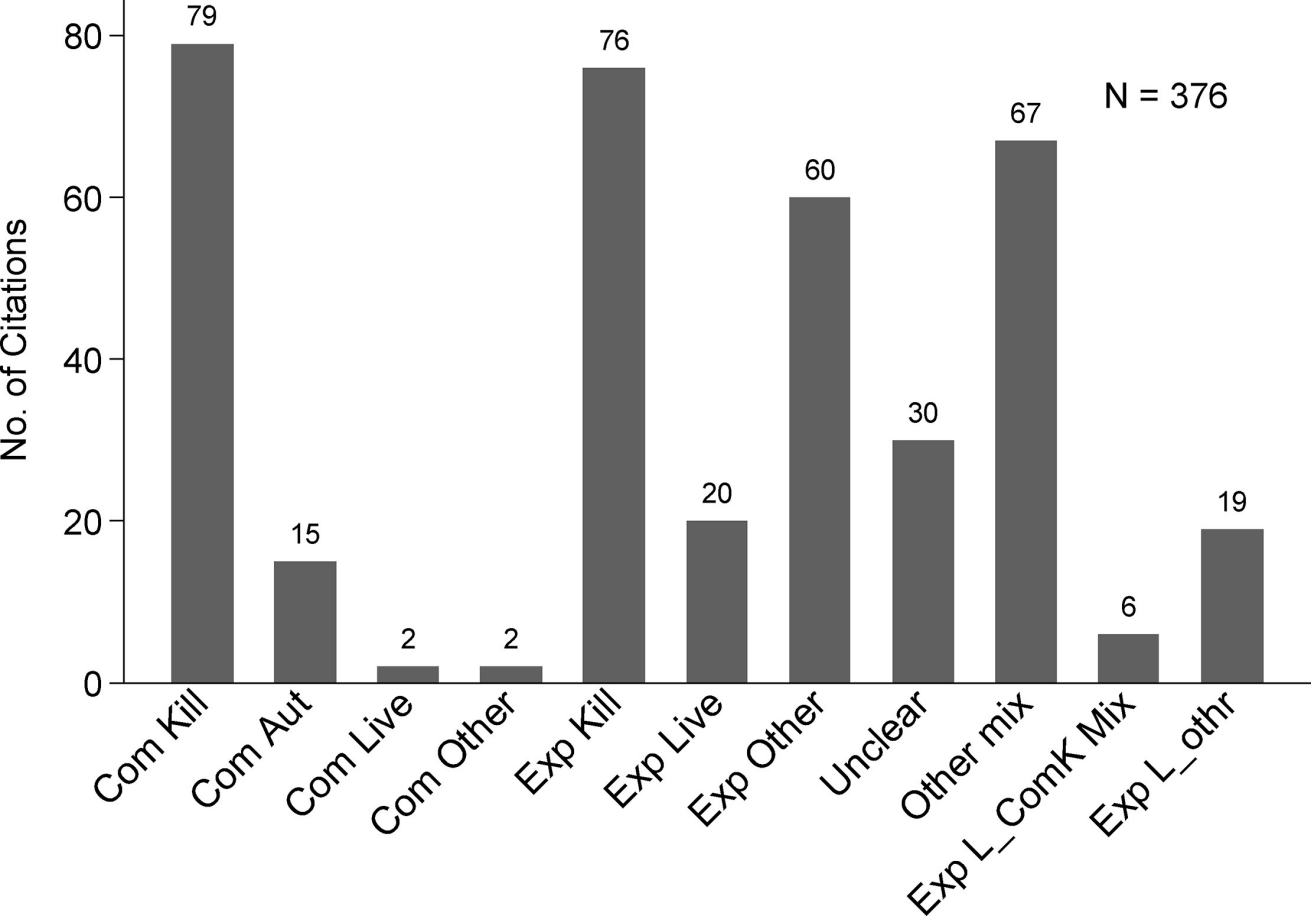
Number of IAV-S vaccine research citations by vaccine type or combination of vaccines reported. Com = Commercial; Exp = Experimental (Kill = killed/inactivated; Aut = autogenous; Live = live; Other = sub-unit, particle, DNA, or non-IAV platform recombinant);Unclear = insufficient details reported to chart vaccine type; Other mix = use of more than one vaccine type reported excluding live vaccines; ExpL_ComK Mix = reported use of both experimental live and commercial killed; Exp L_othr = reported use of experimental live and another vaccine type excluding commercial killed.

**Fig 5:**
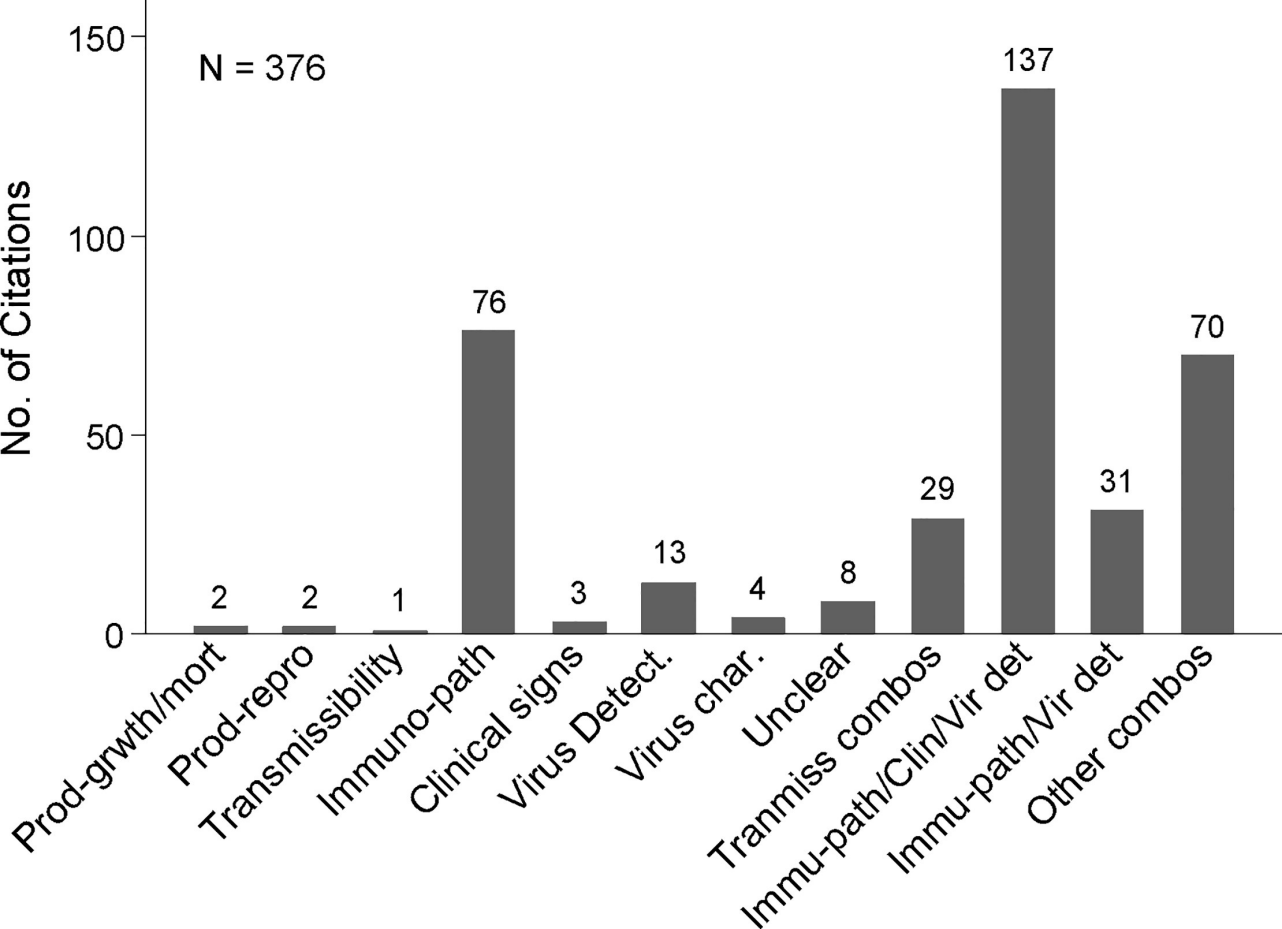
Number of IAV-S vaccine research citations by type of outcome measure or combination of measures reported. Prod-growth/mort = swine production measures of growth (e.g. average daily gain) or mortality; Prod-repo = swine production measures of reproduction (e.g. born-alive, farrowing rate);Transmissibility = measures of viral transfer from one pig to infect another; Immuno-path = host immunologic response to vaccination and viral infection (including lung lesions);Clinical signs = fever, cough, dyspnea; Virus Detect. = detection of virus in samples from pigs; Virus char. = Serotyping and genetic sequencing of virus or other means of typing and subtyping viruses; Unclear = other or unclear where insufficient information was provided to determine outcome measured; Transmiss combos = reporting transmissibility in combination with any other outcome measure; Immu-path/Clin/Vir det = reporting immunopathologic outcomes, clinical signs, and virus detection; Immu-path/Vir det = reporting immunopathologic outcomes and virus detection; Other combos = any combination of reported outcome measures not captured in the other groupings.

Ten characteristics were charted for each of the 376 publications and are summarized by publication type in Table 3. Highlights for each are as follows:

1. **Concurrent interventions:** Concurrent implementation of non-vaccine IAV-S management interventions, applied to either the overall study population or to one study group versus another, was reported in few publications (8%, 31/376).
2. **Study design type:** Experimental hypothesis testing study designs were reported in 92% (157/170) of journal articles and three quarters of conference proceedings (131/175). Observational studies (7%, 27/376) or mathematical modeling vaccinated populations [computer simulation] (3%, 11/376) were reported in few publications.
3. **Comorbidity:** Study population comorbidity with PRDC associated pathogens was reported in few publications (11%, 42/376), the majority of which were conference proceedings (28/376).
4. **IAV exposure type:** The most frequently reported type of population IAV-S virus exposure (233/376, 62%) was challenge trials and was the type reported in almost three quarters of all journal articles (74%, 125/170). Natural virus exposure (i.e. under field conditions) was reported in less than a fifth of publications (19%, 73/376) and mostly in conference proceedings (30%, 53/175) versus journal articles (9%, 15/170). Research reported in almost a fifth of all publications (19%, 71/376) did not involve exposure of the study population to IAV-S virus.
5. **Vaccine type:** Research publications on all vaccine types increased in the early 2000s and then more dramatically after 2009 (Figs 2 and 3). Two thirds of publications involved research on a single type of vaccine (67%, 252/376). Reported details were insufficient to determine the vaccine type involved in the research in 11% of publications (41/376) (Fig 4). Overall, research on killed vaccines was most frequently reported (71%, 267/376) involving almost equally experimental killed (33%, 125/376) or commercial killed (38%, 142/376) vaccines. Research on commercial live vaccines has been sparsely published (1%, 2/376). Research involving [commercial] autogenous vaccines was more frequently reported in conference proceedings (27/175, 15%) than journal articles (7/170, 4%). Eighty-nine percent of research reported in journal articles involved experimental vaccines (152/170).
6. **Vaccinated populations:** Weaned pigs were the most frequently reported population vaccinated in 88% (136/170) of all journal articles and just over half of conference proceedings (51%, 89/175). Almost a quarter of all publications (24%, 89) involved vaccination of breeding herd populations. Publications involving vaccination of gilts were predominantly conference proceedings (88%, 16/20). The type of population vaccinated was categorized as ‘unclear’ for 12% (45/376) of publications, of which most were conference proceedings (80%, 36/45).
7. **Outcome measures:** Overall, the category of immunologic and immunopathologic measures (inclusive of lung scores) was the most frequently reported type of outcome (86%, 323/376) followed by virus detection (65%, 246/376) and clinical signs (54%, 202/376). Production metrics, such as growth performance, feed conversion, or mortality, were infrequently reported (9%, 32/376) as were reproductive performance measures (e.g. farrowing rate, avg. born alive, etc.), (2%, 6/376) (Fig 5). More than one category of outcome measure was reported in almost three quarters (71%, 267/376) of all publications with the combination of immunopathologic, clinical signs, and virus detection jointly reported in over a third of publications (36%,135/376) (Fig 5).
8. **Primary Author Affiliations:** Overall, most primary authors ‘positions or employment were affiliated with universities (53%, 200/376) followed by allied industry (26%, 97/376) and government (22%, 83/376). Forty-five percent of proceedings (78/175) were authored by allied industry affiliates. Over two thirds (69%, 118/170) of journal article primary authors were affiliated with universities.
9. **Region/Country Affiliations:** Most primary authors were affiliated geographically with the U.S.A. (64%, 241/376) followed by the EU (24%, 90/376) and Canada (5%, 20/376). Dissertation authors were almost exclusively university affiliated (96%, 24/25), and most geographically with the U.S. A. (20/25, 80%). The grey literature search included conference proceedings mostly from events held in the U.S.A and was similarly reflected in high author affiliation with the U.S.A. for proceedings (75%, 131/175).
10. **Funding Source:** The research funding source was not stated for half (50%, 187/376) of all publications, the bulk of which were conference proceedings (78%, 145/187). About half of the proceedings with unidentified sources of funding (52%, 75/145) were authored by allied industry and were mostly presented during the AASV Annual Meeting in the session dedicated to industrial partners. Research presented in these sessions may be implicitly understood as industry funded but unless stated explicitly, the publication was charted as ‘unclear’. Excluding these, government was otherwise the most reported source of funds (44%, 165/376), particularly for research published in journal articles (72%, 122/170).

**Table 3:** Counts and percentages by document type of charted IAV-S vaccine in-pig research characteristics.

	Journal Articles		Conference Proceedings		Thesis/ Dissertations		Other Primary Research		Overall Total	
	N = 170		N = 175		N = 25		N = 6		N = 376	
No. of publications	No.	(%)	No.	(%)	No.	(%)	No.	(%)	No.	(%)
**Intervention inclusions**										
Vaccine only	155	(91)	158	(90)	22	(88)	5	(83)	340	(90)
Vaccine plus₮	15	(9)	12	(7)	3	(12)	1	(17)	31	(8)
Unclear	0		6	(3)	0		0		6	(2)
^**◊**^**Study Design Type**										
Hypothesis testing										
Experimental	157	(92)	131	(75)	22	(88)	5	(83)	315	(84)
Observational	5	(3)	19	(11)	3	(12)	0		27	(7)
Computer simulation	5	(3)	4	(2)	2	(8)	0		11	(3)
Descriptive	2	(1)	20	(11)	0		2	(33)	24	(6)
Unclear	1	(1)	2	(1)	0				3	(1)
**Comorbidity**										
Not considered	157	(92)	140	(80)	23	(92)	5	(83)	325	(86)
PRDC considered	11	(6)	28	(16)	2	(8)	1	(17)	42	(11)
Unclear	2	(1)	7	(4)	0				9	(2)
^**◊**^**IAV virus exposure type**										
Challenge	125	(74)	86	(49)	20	(80)	2	(33)	233	(62)
Natural	15	(9)	53	(30)	4	(16)	1	(17)	73	(19)
No Exposure	32	(19)	30	(17)	6	(24)	3	(50)	71	(19)
Unclear	0		11	(6)	0		0	(0)	11	(3)
^**◊**^**Vaccine type**										
Commercial Killed	47	(28)	84	(48)	7	(28)	4	(67)	142	(38)
Commercial Autogenous	7	(4)	27	(15)	1	(4)	1	(17)	36	(10)
Commercial Otherⱡ	2	(1)	1	(1)	1	(4)	0		4	(1)
Commercial Live	0		2	(1)	0		0		2	(1)
**Total Commercial**	**56**	**(33)**	**114**	**(65)**	**9**	**(36)**	**5**	**(83)**	**184**	**(49)**
Experimental Killed	68	(40)	48	(27)	6	(24)	3	(50)	125	(33)
Experimental Otherⱡ	52	(31)	23	(13)	9	(36)	0		84	(22)
Experimental Live	32	(19)	8	(5)	5	(20)	0		45	(12)
**Total Experimental**	**152**	**(89)**	**79**	**(45)**	**20**	**(80)**	**3**	**(50)**	**254**	**(68)**
Unclear	9	(5)	28	(16)	3	(12)	0		40	(11)
^**◊**^**Population type vaccinated**										
Weaned pig	136	(80)	89	(51)	20	(80)	2	(33)	247	(66)
Breeding Herd	26	(15)	56	(32)	6	(24)	1	(17)	89	(24)
Unclear	7	(4)	36	(21)	0		2	(33)	45	(12)
Grower-Finisher	15	(9)	13	(7)	1	(4)	2	(33)	31	(8)
Gilts only (GDU)	3	(2)	16	(9)	1	(4)	0		20	(5)
Neonatal	6	(4)	6	(3)	0		0		12	(3)
Other	1	(1)	0		1	(4)	0		2	(1)
^**◊**^**Outcome type measured**										
Immunopathologic^‡^	162	(95)	133	(76)	24	(96)	4	(67)	323	(86)
Virus detection^‡‡^	130	(76)	98	(56)	18	(72)	0		246	(65)
Clinical signs^‡‡‡^	107	63	75	43	18	72	2	(33)	202	54
Grow-Finish metrics^†^	14	8	13	7	5	20	0		32	9
Transmissibility^††^	15	9	12	7	3	12	0		30	8
Virus characterization^†††^	9	(5)	16	(9)	2	(8)	0		27	(7)
Unclear	4	(2)	11	(6)	0		1	(17)	16	(4)
Reproduction metrics	0		6	(3)	0		0		6	(2)
^**◊**^**Primary Author Affiliation**										
University	118	(69)	56	(32)	24	(96)	2	(33)	200	(53)
Allied Industry	16	(9)	78	(45)	1	(4)	2	(33)	97	(26)
Governmental	57	(34)	24	(14)	0		2	(33)	83	(22)
Unclear	0		8	(5)	0		0		8	(2)
Independent Professional	0		8	(5)	0		0		8	(2)
Production Company	0		3	(2)	0		0		3	(1)
NGO	3	(2)	0		0		0		3	(1)
^**◊**^**Primary Author Country**										
USA	88	(52)	131	(75)	20	(80)	2	(33)	241	(64)
EU	56	(33)	29	(17)	2	(8)	3	(50)	90	(24)
Canada	14	(8)	3	(2)	3	(12)	0		20	(5)
South Korea	3	(2)	4	(2)	0		1	(17)	8	(2)
Unclear	0		8	(5)	0				8	(2)
China	6	(4)	0		0				6	(2)
Other Asian Countries	4	(2)	0		0				4	(1)
Brazil	2	(1)	0		0				2	(1)
Mexico	1	(1)	1	(1)	0				2	(1)
Other Central/South America	0		1	(1)	0				1	
^**◊**^**Funding Source(s)**										
Unclear	28	(16)	145	(83)	12	(48)	2	(33)	187	(50)
Governmental	123	(72)	26	(15)	13	(52)	3	(50)	165	(44)
Allied Industry	27	(16)	9	(5)	5	(20)	0		41	(11)
University	22	(13)	4	(2)	6	(24)	0		32	(9)
International Governmental	9	(5)	0		0		1	(17)	10	(3)
NGO	8	(5)	0		0		0		8	(2)

^**◊**^Publication percentages and counts may exceed 100% and N, respectively, as charted publication can be assigned to more than one category (see also S3 Table, Table 2 for definitions)

₮ Vaccine plus = vaccine intervention applied concurrently with another IAV-S non-vaccine intervention; PRDC = porcine respiratory disease complex (two or more commonly occurring respiratory pathogens involved concurrently in swine herds resulting in clinical respiratory disease)

ⱡOther vaccines = sub-unit, particle, DNA, or recombinant vaccine platform not using an IAV virus RNA backbone

^‡^Immunologic, pathologic, or pathophysiologic responses of the host

^‡‡^ Detection of shedding, or presence of virus (e.g. PCR, IHC)

^‡‡‡^Fever, cough, dyspnea, nasal discharge

^†^Mortality, FC, ADG

^††^Transfer of virus from one pig to another

^†††^Sequencing or antigenic sub-typing.

#### Joint distribution of reported characteristics

Study characteristics are cross-tabulated in Tables 4–6 showing joint distributions, with the highest frequency cells shaded red, and the lowest blue. Vaccination of weaned pig populations with killed vaccines, use of challenge study designs, and measurement of immunologic/immunopathologic outcomes (Tables 4–6), were the most frequent combinations of study characteristics reported in publications. Challenge trials involving vaccinated weaned pigs were reported in almost half of all publications (49%, 185) versus considerably fewer challenge trials involving vaccination of breeding herd populations (9%, 34/376) (Table 4). Across all population types, the most frequently reported outcomes were immunologic or immunopathologic responses, followed by virus detection, and clinical signs (Table 5). The type of vaccine used to vaccinate breeding herds was unclear in almost a quarter of the relevant publications (22/89) (Table 6). The percentage of publications involving vaccinated breeding herds under conditions of natural exposure (54%, 48/89), exceeded that of publications involving vaccinated weaned pigs under similar exposure conditions (6%, 14/247) (Tables 3 and 4). Publications involving research of experimental vaccine under conditions of natural virus exposure were rare (2%, 7/376) (Table 6). Publications involving experimental live vaccine research and either transmissibility (3/376) or virus characterization (1/376) were also rare (Table 6).

**Table 4:** Heat map chart of frequencies and overall percentages of publications jointly reporting type of populations vaccinated and outcome measure reported, by type of virus exposure.

Virus Exposure Type	Challenge		Natural Exposure		No Virus Exposure		Unclear	
	No.	%	No.	%	No.	%	No.	%
**Population vaccinated**								
Weaned pigs	185	49	14	4	53	14	2	1
Breeding Herd Females	34	9	48	13	9	2	5	1
Unclear	28	7	6	2	7	2	6	2
Grower/Finisher	9	2	17	5	7	2	0	0
Neonatal piglets	8	2	0	0	4	1	0	0
Gilts in development programs	1	0	18	5	1	0	0	0
Other	0	0	2	1	0	0	0	0
**Outcome Measure Reported**								
Immunopathologic^†^	221	59	38	10	68	18	7	2
Virus detection^††^	206	55	41	11	5	1	3	1
Clinical signs^†††^	171	45	20	5	17	5	2	1
Transmissibility^‡‡‡^	25	7	7	2	1	0	0	0
Production parameters- growth^‡^	18	5	8	2	7	2	1	0
Virus characterization^‡‡^	9	2	19	5	0	0	1	0
Unclear or other	3	1	10	3	1	0	3	1
Production parameters—reproduction	0	0	4	1	0	0	3	1

^†^Immunologic, pathologic, or pathophysiologic responses of the host

^††^Detection of virus shedding or presence of infection (e.g. PCR, IHC)

^†††^Fever, cough, dyspnea, nasal discharge

^‡^Mortality, FC, ADG

^‡‡^Genomic sequencing or antigenic sub-typing

^‡‡‡^Transmission of virus from one pig to another. Publication characteristic is listed in **bold type**; highest frequencies are in cells shaded red and the lowest in blue; % = number of publications in the cell (tandemly reporting categories) divided by the overall total publication count (N = 376). Overall percentages for each characteristic will exceed 100% as it possible to chart a publication to more than one category per type of characteristic (see also S4 Table, Table 2 for definitions).

**Table 5:** Heat map chart of frequencies and overall percentages of publications jointly reporting outcome measures, by population type vaccinated.

Population Type Vaccinated	Gilts		Breeding Herd		Neonatal		Weaned pigs		Grow/Finisher		Other		Unclear	
	No.	%	No.	%	No.	%	No.	%	No.	%	No.	%	No.	%
**Outcome Measure Reported**														
Immunopathologic^†^	10	3	60	16	11	3	253	67	23	6	0	0	35	9
Virus detection^††^	13	3	59	16	8	2	176	47	16	4	1	0	21	6
Clinical signs^†††^	10	3	38	10	7	2	153	41	17	5	0	0	14	4
Production parameters-growth^‡^	1	0	7	2	0	0	23	6	3	1	0	0	2	1
Transmissibility^‡‡‡^	2	1	13	3	0	0	13	3	2	1	0	0	4	1
Virus characterization^‡‡^	7	2	10	3	0	0	10	3	5	1	1	0	5	1
Unclear or other	4	1	9	2	0	0	2	1	4	1	0	0	4	1
Production parameters-reproduction	1	0	6	2	0	0	1	0	0	0	0	0	0	0

^†^Immunologic, pathologic, or pathophysiologic responses of the host

^††^Detection of virus shedding or presence of infection (e.g. PCR, IHC)

^†††^ Fever, cough, dyspnea, nasal discharge

^‡^Mortality, FC, ADG

^‡‡^Genomic sequencing or antigenic sub-typing

^‡‡‡^Transmission of virus from one pig to another. Publication characteristic is listed in **bold type**; highest frequencies are in cells shaded red and the lowest in blue; % = number of publications in the cell (tandemly reporting categories) divided by the overall total publication count (N = 376). Overall percentage for each characteristic will exceed 100% as it possible to chart a publication to more than one category /characteristic (see also S4 Table, Table 2 for definitions).

**Table 6:** Heat map chart of frequencies and overall percentages publications jointly reporting population vaccinated, virus exposure, and outcome measure, by type of vaccines used.

Vaccine Type	Commercial								Experimental							
	Killed		Autogenous		Live		Other ^Ŧ^		Killed		Live		Other ^Ŧ^		Unclear	
	No.	%	No.	%	No.	%	No.	%	No.	%	No.	%	No.	%	No.	%
**Population vaccinated**																
Weaned pigs	96	26	11	3	0	0	3	1	92	24	36	10	70	19	7	2
Breeding Herd Females	45	12	20	5	0	0	1	0	20	5	7	2	7	2	22	6
Unclear	16	4	5	1	0	0	0	0	18	5	7	2	7	2	6	2
Grower/Finisher	9	2	5	1	0	0	1	0	6	2	1	0	8	2	8	2
Gilts in development programs	6	2	9	2	0	0	2	1	0	0	0	0	4	1	5	1
Neonatal piglets	5	1	0	0	2	1	0	0	1	0	2	1	2	1	3	1
Other	0	0	0	0	0	0	0	0	0	0	0	0	0	0	2	1
**Viral Exposure**																
Challenge	83	22	7	2	2	1	2	1	94	25	41	11	65	17	12	3
Natural Exposure	34	9	27	7	0	0	2	1	4	1	1	0	3	1	24	6
No Virus Exposure	23	6	2	1	0	0	0	0	28	7	5	1	19	5	2	1
Unclear	4	1	1	0	0	0	0	0	2	1	0	0	1	0	4	1
**Outcome Measure Reported**																
Immunopathologic^†^	121	32	22	6	1	0	3	1	117	31	44	12	81	22	19	1
Virus detection^††^	90	24	22	6	2	1	3	1	82	22	38	10	63	17	22	1
Clinical signs^†††^	70	19	10	3	2	1	2	1	71	19	31	8	56	15	13	1
Production parameters- growth^‡^	15	4	1	0	0	0	0	0	6	2	0	0	9	2	5	1
Virus characterization^‡‡^	14	4	13	3	0	0	0	0	3	1	1	0	3	1	4	1
Transmissibility^‡‡‡^	12	3	4	1	0	0	0	0	16	4	3	1	2	1	2	1
Production parameters–reproduction	5	1	1	0	0	0	0	0	1	0	0	0	0	0	0	1
Unclear or other	4	1	5	1	0	0	0	0	2	1	1	0	3	1	9	1

^Ŧ^ Other includes sub-unit, particle, DNA, or recombinant vaccine platform which is not an IAV virus RNA backbone

^†^Immunologic, pathologic, or pathophysiologic responses of the host

^††^Detection of virus shedding or presence of infection (e.g. PCR, IHC)

^†††^ Fever, cough, dyspnea, nasal discharge

^‡^Mortality, FC, ADG

^‡‡^Genomic sequencing or antigenic sub-typing

^‡‡‡^Transmission of virus from one pig to another. Publication characteristic is listed in **bold;** highest frequencies are in cells shaded red and the lowest in blue; % = number of publications in the cell (tandemly reporting categories) divided by the overall total publication count (N = 376). Overall percentages for each characteristic will exceed 100% as it possible to chart a publication to more than one category per type of characteristic (see also S4 Table, Table 2 for definitions).

Investigation of transmissibility was limited overall (8%, 30/376) (Table 3) and involved most often use of challenge trials (83%, 25/30) (Table 4), commercial killed (40%, 12/30) or experimental kill vaccines (53%, 16/30) (Table 6), in weaned pig (40%, 12/30) or in breeding herd populations (43%, 13/30) (Table 5). Investigation of virus characterization was also limited and involved mostly use of commercial killed (n = 14) or autogenous vaccines (n = 13) (Table 6), under conditions of natural virus exposure (n = 19) (Table 4), across several different types of vaccinated populations (Table 5).

#### Identifying potential combinations of elements for systematic review questions

Nineteen different combinations of elements were selected as potential review questions based on the highest frequency cells in Tables 4, 5 and 6. Counts of publications matching each combination are summarized by publication type in Table 7. Most publications involving natural virus exposure of vaccinated breeding herd populations (75% (36/48)) or of vaccinated weaned pigs (71% (10/14)) were conference proceedings (See lines 5 & 13 in Table 7). Conversely, of the 112 journal articles involving vaccinated weaned pigs exposed to virus, less than 3% (n = 3) reported the exposure as natural (Table 7, line 13). Journal articles involving exposure of vaccinated breeding herds to virus similarly included proportionally fewer reports, as compared to proceedings, where the virus exposure was natural (36%, 9/25) (Table 7, lines 5&6). All publications involving breeding herds vaccinated with autogenous vaccines and exposure to virus, reported only natural virus exposure (i.e. no challenge trials) (Table 7, lines 10&19).

**Table 7:** Counts of IAV-S swine vaccine publications matching selected PICO^Ŧ^ components for potential systematic review questions.

Line	Population	Intervention (Vaccine type)		Virus exposure		Comparator & Outcomes		No. of Publications†		Conf. Proc.	Journal Articles
1	Weaned Pigs	&	All	&	Challenge	&	All	=	185	59	109
2	Weaned Pigs	&	Killed Experimental	&	Challenge	&	All	=	71	23	46
3	Weaned Pigs	&	Killed Commercial	&	Challenge	&	All	=	70	35	29
4	Weaned Pigs	&	Experimental Other	&	Challenge	&	All	=	57	12	37
5	Breeding herd	&	All	&	Natural Exposure	&	All	=	48	36	9
6	Breeding herd	&	All	&	Challenge	&	All	=	34	12	16
7	Breeding herd	&	Killed (commercial and experimental)	&	Challenge	&	All	=	29	11	14
8	Breeding herd	&	Killed (commercial and experimental)	&	Natural Exposure	&	All	=	26	18	6
9	Breeding herd	&	Killed Commercial	&	Natural Exposure	&	All	=	23	16	5
10	Breeding herd	&	Autogenous	&	Natural Exposure	&	All	=	20	17	2
11	Breeding herd	&	Killed Experimental	&	Challenge	&	All	=	17	4	10
12	Breeding herd	&	Killed Commercial	&	Challenge	&	All	=	15	7	6
13	Weaned Pigs	&	All	&	Natural Exposure	&	All	=	14	10	3
14	Weaned Pigs	&	Killed Commercial	&	Natural Exposure	&	All	=	8	4	3
15	Weaned Pigs	&	Autogenous	&	Challenge	&	All	=	5	2	3
16	Weaned Pigs	&	Autogenous	&	Natural Exposure	&	All	=	4	4	0
17	Breeding herd	&	Killed Experimental	&	Natural Exposure	&	All	=	3	2	1
18	Weaned Pigs	&	Commercial Other	&	Challenge	&	All	=	2	0	2
19	Breeding herd	&	Autogenous	&	Challenge	&	All	=	0	0	0
20	Gilt Development	&	All	&	Challenge	&	All	=	0	0	1
21	Gilt Development	&	All	&	Natural Exposure	&	All	=	18	15	2
22	All	&	All	&	Natural Exposure	&	TM^‡^	=	7	3	3
23	Weaned Pigs	&	All	&	Natural Exposure	&	TM^‡^	=	1	0	1
24	Breeding herd	&	All	&	Natural Exposure	&	TM^‡^	=	5	2	2
25	All	&	All	&	Natural Exposure	&	VC^‡‡^	=	19	13	4
26	Weaned Pigs	&	All	&	Natural Exposure	&	VC^‡‡^	=	5	4	0
27	Breeding herd	&	All	&	Natural Exposure	&	VC^‡‡^	=	9	6	2

^Ŧ^PICO = Population (P), Intervention (I), Comparator (C), and Outcome (O), are the question components for systematic reviews of interventions

^†^Publications may fit with more than one combination of components (see also S4 Table, Table 2 for definitions), and include journal articles, conference proceedings, theses/dissertations, and ‘other’ primary research documents; Conf. Proc. = conference proceedings

^‡^TM = Transmissibility

^‡‡^VC = Virus Characterization. Individual counts by publication type are shown only for journal articles and conference proceedings.

#### Important potential gaps in research

*Research on autogenous vaccines in weaned pigs under field conditions*. Reports of [commercial] autogenous vaccine research in weaned pig populations includes 9 publications, 5 as challenge trials, (of which 3 are journal articles), and 4 under conditions of natural virus exposure (of which none is a journal article) (lines 15&16 of Table 7).

*Field research involving vaccination and transmissibility or virus characterization*. Reports inclusive of virus characterization, or of transmissibility under conditions of natural exposure, were relatively sparse across all types of vaccinated populations and across all publication types (lines 22–27, Table 7). Study of transmissibility in vaccinated weaned pigs under conditions of natural virus exposure was reported in a single journal article (line 23).

*Research on commercial live vaccines under field conditions*. At the time of this review there was no published research on use of commercial live vaccines under conditions of natural virus exposure.

## Discussion

### Summary of evidence

Upkeep of a scoping review or evidence map of IAV-S vaccine research in swine as a current document, using the charting template applied in this review, may help stakeholders understand the frequencies of reported study characteristics, and therefore aid in convergence of priorities for allocation of resources [38]. Foreknowledge of the quantity of relevant publications can help when balancing the scope of a synthesis project with the resources available for the project [39]. In this way, citations lists produced from a scoping review may also be useful for the planning of future and related synthesis projects [40–42].

Four observations were of particular note after mapping the ten selected characteristics:

#### 1. Published weaned pig challenge trials far exceed published field trials

Publication of vaccinated weaned pig challenge trials far exceeded that of field trials despite the relatively long history and broad use of licensed IAV-S vaccines in growing pig populations. There are concerns with undue reliance on research from challenge trials to inform vaccine program decisions for the field. A pig’s first IAV exposure impacts immunologic responses to all subsequent exposures both positively and negatively; a phenomenon referred to as ‘original antigenic sin’ [43, 44]. Outcomes reported in challenge trials, where prior exposure can be controlled by the researcher, are therefore more likely to be poorly predictive of responses to vaccination in the field where the population’s prior or current viral exposure may not be defined [43, 45]. Additionally comorbidity and ‘multiple hurdle’ approaches to manage disease (where more than one intervention is employed concurrently during a production cycle) are common in modern swine production and can also potentially impact the effectiveness of a vaccine program [21, 46–51]. The impact of either is difficult to assess using challenge trials. Challenge trials are useful, for example, to validate correlates of protection, to assess vaccine efficacy for licensure, or to confirm emergence of viral resistance to commercial IAV-S vaccines [52–55], but ultimately field studies conducted in multiple settings, using appropriately sized study populations, or both, are needed for the evaluation and on-going assessment of IAV-S vaccines as an effective tool for influenza control in swine populations [38, 52, 56–58]. Here we differentiate vaccine efficacy or measures of protection for the vaccinated individual, from vaccine effectiveness, an evaluation of how well a vaccine program is protecting swine herds in the field [38, 59, 60]. Research under field conditions was limited for commercial killed vaccines, rare for commercial autogenous vaccines, and not available for commercial live vaccines. This suggests possible important gaps in a publicly available record of IAV-S vaccine program effectiveness [38, 53, 61]. It is also possible however that field research is actively conducted within larger integrated productions systems but that findings remain ‘in-house’ only. Alternatively, in the absence of publicly available field data, modeling and computer simulation studies can be instructive for the design of subsequent field studies because the exercise of modelling makes explicit current assumptions and gaps in the understanding of factors impacting vaccine effectiveness [22, 55, 62–65]. Publications involving mathematical modelling in IAV-S vaccine research in swine are rare. The reasons for this are unknown [63].

#### 2. More research under field conditions was published in proceedings than in journals

Although most IAV-S vaccine primary research was published almost equally as conference proceedings or journal articles, research involving conditions or practices closely related to the field, and research authored by allied industry, was disproportionately reported in conference proceedings versus journal articles. This suggests research disseminated through conferences may differ from that in journal articles. Additionally, during our search we identified different issues with access to proceedings than for journals. The majority of proceedings were not catalogued in bibliographic databases, were available to members only, and were inconsistently accessible on professional association websites [66]. Conversely, relevant journal articles were published across fifty-one different academic journals of which most were not an open access format. Full-text access to non-open access journal articles might then be prohibitively restrictive for veterinarians without institutional or other organizational subscriptions to bibliographic databases or e-journals [67]. Lam et al. [68] reported that message continuity is important for effective implementation of disease control interventions. It is therefore a reasonable concern that content and access differences might contribute to inconsistent communication of IAV-S vaccine information in the field.

#### 3. Production metrics were seldom reported as outcomes

Vaccination in swine populations is primarily an economically driven decision. Although primary author geographic affiliations coincided with the world’s top pork exporting regions (EU, USA, Canada, and Brazil [69]), there was relatively little emphasis on reporting of production outcomes (i.e. feed conversion, average daily gain, farrowing rate, average weaning weight, etc.). Instead, immunologic and immunopathologic outcomes, virus detection, and clinical signs, were reported most frequently, as consistent with the reporting of influenza vaccine research in other species [16, 70, 71].

#### 4. Very little vaccine research included study of transmissibility or of genomic sequencing

In 2014, OFFLU (OIE/FAO network of expertise on animal influenza) outlined a global strategic agenda for animal influenza research with the assertion that ‘genetic sequence data will play a central role in predictive understanding in IAV risk management’ [17]. In both vaccinated and non-vaccinated pigs, influenza A viruses can be diverse at the level of the population, within an individual pig, and even within a cell [72–75]. Investigation of virus characterization and of transmissibility is necessary to understand, at the individual animal level, vaccine induced cross-protection and duration of immunity, and at the population level, the impact of vaccination on the viral ecology of influenza in swine [1, 21, 56, 76–82]. In poultry, vaccination has been associated with selective transmission of more virulent strains of IAV [83]. IAV-S vaccine research in pigs conducted under conditions of natural exposure and involving study of transmissibility, or virus characterization was rarely published in journal articles. This raises the concern that understanding of the role of vaccine immune pressure on the stability or emergence of new variants of IAV-S may similarly be limited [22, 72, 73, 84–90].

## Future considerations and next steps

It may be possible to address limitations of this scoping review using systematic review methods [68, 91–95]. This review did not assess risks of bias, identify repeated reporting of results in different categories of publications, or provide a detailed accounting of research methodologic heterogeneity.

The contribution of conference proceedings to the broader evidence ecosystem as an alternate or an abridged reporting of research can be significant [6, 66, 96]. Assessment of risks of bias and analysis of publication trends has provided additional insight on the role of proceedings in veterinary vaccine research as a knowledge translation resource [97, 98] Similar analysis of IAV-S proceedings may improve understanding of their contribution to the body of research evidence.

Six questions for potential systematic review of IAV-S vaccine research in pigs are proposed in Table 8. Just as larger sample sizes can improve precision and accuracy, it is preferable for a body of relevant research to be sufficiently large when conducting a systematic review.

Question elements (population, intervention, and outcome) were therefore selected based also on frequencies of jointly reported characteristics (Table 7). Four additional assumptions were also considered in the selection of proposed questions: 1) Breeding herd infectious disease control is prerequisite for control of infection in the larger population [34, 99] therefore, assessment of vaccine-induced protection in the breeding herd was a presumed industry priority. 2) The seven categories used to chart outcome data were insufficient to convey the diversity of reported outcomes measures. 3) Outcome diversity is predominantly and importantly a function of the researchers‘ differing conceptual definitions of IAV-S vaccine-induced protection in swine, and a function of the quickly evolving technical understanding of IAV-S in swine [15, 70, 74, 100–105]. 4) Research is likely insufficient to support systematic review of a narrowly defined outcome given the diversity of viruses involved, and the importance of strain specificity for immunologic responses. Rather, a broader yet potentially more informative objective is to make explicit the diversity of conceptual definitions of protection, and to define, and synthesize where possible, methodologic heterogeneity for the reported measures of protection [103]. A specific measureable outcome was therefore not picked, and instead the umbrella term ‘protection’ was used in all questions.

**Table 8:** Six questions ^Ŧ^ for potential systematic review of IAV-S vaccine research and counts of available relevant publications.

Potential systematic review question	No. CP	No. JA	No. T/D	No. O	Tot. No.	Table 7 ref.
1. What protection^†^ against natural IAV-S virus exposure is provided by vaccinating breeding herds with commercial killed vaccines?	16	5	1	1	23	Line 9
2. What protection^†^ against natural IAV-S virus exposure is provided by vaccinating breeding herds using any vaccine type	36	9	9	2	48	Line 5
3. What protection^†^ against natural IAV-S virus exposure is provided by vaccinating breeding herds with autogenous vaccines	17	2		1	20	Line 10
4. What protection^†^ against natural IAV-S exposure is provided by vaccinating gilts in development units	15	2	1		18	Line 21
5. Do IAV-S commercial killed vaccines protect^†^ weaned pigs when challenged with IAV-S virus?	35	29	6		70	Line 3
6. What protection^†^ against natural IAV-S virus exposure is provided by vaccinating weaned pigs?	10	3	1		14	Line 9

^Ŧ^ Question components were selected using the informal criteria of 1) an assumed industry prioritization for synthesis of research on vaccination of sow herd populations and of commercial vaccine performance in the field, 2) potentially relevant publications could be identified in numbers sufficient to support a systematic review, and 3) questions needed to be broadly inclusive due to expectations of high methodologic heterogeneity in outcome measurement and reporting

† all reported outcomes were included under the umbrella term of ‘protection’

^††^other = other type of primary research document; CP = Conference proceeding; JA = Journal articles; T/D = Theses or dissertation; O = other type of primary research publication; Tot. No. = total number of publications matching selected PICO components in question.

## Limitations

There were several limitations of this review. Data items for study comparator groups were not charted. Data was charted by a single reviewer (the primary author) increasing the potential for information bias [31]. The reviewer, however had also screened all citations at least three times for relevance prior to charting, and we believe this familiarity with the evidence helped to mitigate this risk. *A priori* the 10 characteristics and category options were selected to be clearly definable and objectively charted. Distinguishing outcomes of virus characterization from virus detection was, however, complicated as modern commercially available IAV-S diagnostic assays for virus detection frequently also include some information on virus characterization [106]. Outcomes were charted as virus characterization if the researcher’s end objective was interpreted as investigation of lineages and/or subtypes of circulating viruses within an animal or population post-vaccination. It is possible that publications reporting outcomes of virus characterization may have been charted as virus detection in error.

Exclusion of proceedings lacking digitized metadata also biased selection towards those from the AASV online Library which catalogues mostly conferences held in the U.S.A. Although identical citations were eliminated, the final publication yield is inflated because duplicative publishing of the same research items, or components of the same research in a different type or types of publications, was not assessed. For example, it is reasonable to assume research included in the 25 relevant theses and dissertations were likely also published as a proceeding (s) and/or journal article(s), yet all publications were included if relevant.

Characteristics were charted at the level of the publication, not at the level of the study. If multiple trials were reported in a publication, all reported characteristics were cumulatively charted as a single publication. It is possible that publications not including the terms vaccination, immunization, or other vaccine related terms in either the title or abstract may have been excluded in error during relevance screening. Although it is possible that vaccine research was published elsewhere and not included due to our inclusion criteria, the original search was broadly inclusive and entirely screened for relevance before the scope was narrowed to vaccine research. Having screened first this wider body of evidence, we believe our search was sufficiently comprehensive. We do not believe there were other significant sources of bias in this review.

## Conclusions

IAV-S vaccine research was published across multiple journals and conference proceedings where access was largely limited to subscribers, and to association members, respectively. Vaccination of weaned pig populations with killed vaccines, use of challenge study designs, and measurement of immunologic/immunopathologic outcomes were the most frequent combinations of study characteristics reported in publications. Publication of research in vaccinated breeding herd populations, the production stage most targeted for vaccination in the U.S.A. [107] was modest. Research involving vaccinated weaned pigs under conditions of natural virus exposure, or weaned pigs vaccinated with autogenous vaccines, was sparse, whereas in practice, autogenous vaccines is the vaccine type used almost exclusively in U.S. growing pigs [107]. Overall, publications are limited of IAV-S vaccine research conducted under field conditions with natural virus exposure, comorbidity associated with PRDC, and/or implementation of concurrent IAV-S control interventions. This suggests there may be important gaps in vaccine effectiveness research. Other identified gaps include research on transmissibility, and on use of live vaccines in the field. Mathematical modelling and computer simulation studies of IAV-S vaccine use in swine populations may be an underutilized approach to vaccine effectiveness research.

As next steps, 6 broadly inclusive questions for potential systematic review were suggested prioritizing study of vaccinated breeding herd populations and the assessment of methodologic heterogeneity.

## Supporting information

S1 Checklist Preferred Reporting Items for Systematic reviews and Meta-Analyses extension for Scoping Reviews (PRISMA-ScR) checklist. (DOCX)

S1 Fig Counts of charted journal articles (n = 170) by journal titles (n = 51). Journals listed on DOAJ (Directory of Open Access Journals) are shown as bars with a dashed outline. (See S6 Table for supporting information).
(DOCX)

S1 Text Relevance screening forms L1, L2, & L3 explanation and elaboration–notes for reviewers. (DOCX)

S1 Table Formatted search strings for Web of Science and CAB direct databases. †Search strategy and search strings were developed and formatted for selected bibliometric platforms with support from University of Guelph librarians with expertise and experience in systematic review methods.
(DOCX)

S2 Table Level 1 (L1) relevance screening form for title/abstract screening. Questions and definitions were pre-tested and refined using a minimum sampling of 100 citations and any conflicts or question were resolved before proceeding with screening of all citations. Forms implemented in Distiller-SR. Citations were forwarded to the next level of screening if reviewed as relevant through sequential asking of all questions on a form, or if relevance was determined as unclear by both reviewers. A third reviewer from the team decided on cases of unresolved reviewer disagreement. See S1 Text for explanatory notes and S5 Table for definitions.
(DOCX)

S3 Table Level 2 (L2) relevance screening form for title/abstract screening. (DOCX)

S4 Table Level 3 (L3) relevance screening and data charting form for full text screening. Pre-testing and refinement of the Level 3 data charting form was done using full text journals articles (n = 21) until charting was consistent between reviewers. Forms implemented in Distiller-SR. Level 3 includes three modified relevance screening questions carried over from Level 1 and Level 2 forms. See S1 Text for explanatory notes and S5 Table for definitions.
(DOCX)

S5 Table Definitions applied in forms 1, 2 & 3 (L1, L2, L3) for relevance screening and data charting. (DOCX)

S6 Table Summary of journal article counts by journal titles and listing status as Open Access (OA) on Directory of Open Access Journals (DOAJ). (DOCX)

S7 Table Conference proceeding counts and conference event counts for conference series, by bibliographic database source^†^. ^†^There was no duplicative indexing of proceedings by CABD and WOS; the AASV Library citation was deduplicated if indexed also in CABD and/or WOS.
(DOCX)

S1 Appendix Citation listing of 376 relevant and characterized citations sorted by title—all data. This Excel file contains 5 worksheets (1) overall data, and summary listings of (2) conference proceedings, (3) journal articles, (4) theses and dissertations, (5) other publications. Each worksheet is sorted by citation title.
(XLSX)
