# Supplementary material for: Influenza A virus vaccine research conducted in swine from 1990 to May 2018: A scoping review
Source: PLoS One. 2020 Jul 16;15(7):e0236062. doi: 10.1371/journal.pone.0236062 (PMC7365442; doi:10.1371/journal.pone.0236062)
Supplement: S1 Fig — Journals listed on DOAJ (Directory of Open Access Journals) are shown as bars with a dashed outline. (See S6 Table for supporting information). (DOCX) [file pone.0236062.s002.docx]

**S1 Fig.** Counts of charted journal articles (n=170) by journal titles (n=51).

Journals listed on DOAJ (Directory of Open Access Journals) are shown as bars with a dashed outline.

(See also S6 Table for supporting information)
