# Supplementary material for: Influenza A virus vaccine research conducted in swine from 1990 to May 2018: A scoping review
Source: PLoS One. 2020 Jul 16;15(7):e0236062. doi: 10.1371/journal.pone.0236062 (PMC7365442; doi:10.1371/journal.pone.0236062)
Supplement: S1 Text — (DOCX) [file pone.0236062.s003.docx]

**S1 Text. Relevance Screening Forms L1, L2, & L3 Explanation and Elaboration – notes for reviewers.**

**Several relevance screening questions were repeated at level 1, 2 and 3 as follows:**

**L1 & L2 Question 1**.

**Is this report/study/document about Influenza A virus in/from swine (IAV-S) where swine or direct applicability to swine is the focus?**

Influenza A virus in/from swine (IAV-S) is also referred to as swine influenza viruses (SIV) but is not to be confused with ‘swine flu’, a misnomer assigned most recently to the 2009 pandemic strain (1).

Influenza A viruses are identified antigenically using serotyping technology based on viral surface proteins hemagglutinin (HA- 18 different subtypes) and neuraminidase (NA -9 different subtypes). Subtypes identified as endemic in swine populations H1N1, H3N2, and H1N2 (occasionally H3N1, H2N3) All citations where the virus was not directly recovered from swine were excluded or reporting IAV-S was in non-swine species only were excluded.

Examples of topics involving both IAV-S and swine included but was not limited to topics of antigenic drift, antigenic shift, research analytics (such as molecular biology, big data, molecular epidemiology, next generation sequencing, omics), virus characterization, development or validation of laboratory methods and diagnostics, pathophysiology, immunology, infection dynamics, transmission, surveillance (and monitoring), phylogeny, non-vaccine risk factors, interventions (protective factors), IAV-S Vaccines (development and evaluation), and costs of disease (economics).

**L1 Question 2.**

**Is the full body text beyond the title/abstract available in English?** – No further explanation.

**L1 Question 3. & L2 Question 2.**

**Is this citation primary research?**

**(**Note: wording differed for **L3 Question 1. What is the document type?)***

Primary research was defined as new findings as published in literature (e.g. in the format of conference proceedings, academic journal articles, theses/ dissertations, etc.)**.** Reviews – (traditional or narrative, systematic reviews with or without a meta-analysis, and meta-analyses), editorial or commentaries, white papers, working reports, policy papers, issue papers, and guidelines were all considered as publications other than primary research.

**L1 Question 4. & L2 Question 3. Is the unit of study exclusively at the sub-animal level (e.g. tissue, cellular, molecular level)?** – No further explanation.

**L3 Question 4. What is the unit of study/ test population or level of study? Select all that apply**

Citations were excluded if the research at no time involved sampling, manipulations, or analysis (either *in vivo* or *in silico*) at the whole pig or higher level (e.g. group, pen, batch, farm, production system, regional, etc.). Research exclusively at the bench top level (*in vitro, ex vivo)* was not relevant for this scoping review.

**L1 Question 5. &L2 Question 4.**

**What kind of review is this document?**

**(L3 Question 1. What is the document type?)***

This question only applied to citations identified as a review in L1 question 3 or in L2 question 2. (L3 question 1 also assigns review types to excluded citations) For the purpose of this scoping review we did not assess if a review met the criteria as per the definition of Cooper and Hedges(2) of a systematic review or meta-analysis , rather, citations where the author identifies at the title or abstract level the review as a ‘systematic review’ and/or a meta-analyses were identified as such. Citations where authors do not describe the review as a ‘systematic review’ will be considered narrative or traditional reviews.

**L2 Question 5.**

**Does this research involve: risk factors or interventions, vaccine development or evaluation, estimation of infection dynamic or transmission parameters?**

Each of these topics is important at the field level in the control of influenza in swine populations and it is not uncommon for a study when focusing on one of these topics to also include focus or for outcomes to be influenced by one or both of the other topics. Included research involves investigation of factors inferred or associated with increasing (risk factor) or decreasing (interventions or protective factors) the risk of IAV-S infection in swine. Interventions included protective factors such as products or programs to reduce the risk or impact of infection and include also study of risk management and biosecurity actions taken to limit exposure, transmission and spread at the animal level or higher (i.e. within production site, between production sites, regions, etc.).Vaccination can be both a risk factor and an intervention. Swine vaccine development and evaluation research at the bench top level or involving trials in non-swine species were not included.

Research on infection dynamics and transmission was also included if it involved discussion specifically about the determination of transmission parameters and infection dynamics at the level of pig-to-pig, within group, within farm, within regions but not pig-to-human transmission.

**L2 Question 6. & L3 Question 2.**

**Does this study involve vaccine evaluation or development trials in swine?**

No further explanation.

(1) 5m Editor. Economic Impact of the “Swine Flu” Misnomer Setember 18, 2009, at 12:00am [Internet]. The Pig Site. 2009 [cited 2019 Feb 2]. Available from: https://thepigsite.com/articles/economic-impact-of-the-swine-flu-misnomer

(2) Cooper H, Hedges L V. Research synthesis as a scientific process. In: Cooper H, Hedges L V., Valentine JC, editors. The handbook of research synthesis and meta-analysis. Second. New York: Russell Sage Foundation; 2009. p. 3–16.
