# Supplementary material for: Influenza A virus vaccine research conducted in swine from 1990 to May 2018: A scoping review
Source: PLoS One. 2020 Jul 16;15(7):e0236062. doi: 10.1371/journal.pone.0236062 (PMC7365442; doi:10.1371/journal.pone.0236062)
Supplement: S1 Table — †Search strategy and search strings were developed and formatted for selected bibliometric platforms with support from University of Guelph librarians with expertise and experience in systematic review methods. (DOCX) [file pone.0236062.s004.docx]

**S1 Table. Formatted search strings**† **for Web of Science and CAB Direct Databases.**

| **Formatted for Web of Science:**  TS=(pork OR swine OR "Sus scrofa" OR pig OR pigs OR piglet OR piglets OR gilt OR gilts OR boar OR boars OR sow OR sows OR hog OR hogs OR “weaner pig” OR “weaned pig$” OR “feeder pig$” OR feeder OR feeders OR “finisher pig$” OR “finisher hog$” OR porcine OR “market-weight” NOT "guinea pig$")  AND TS=(influenza OR IAV OR flu OR SIV OR “H3N2” OR “H1N1” OR “H1N2” OR “H3N1” OR “H2N3”) |
| --- |
| **Formatted for CAB Direct:**  ((pork OR swine OR "Sus scrofa" OR pig OR pigs OR piglet OR piglets OR gilt OR gilts OR boar OR boars OR sow OR sows OR hog OR hogs OR “weaner pig” OR “weaned pig*” OR “feeder pig*” OR feeder OR feeders OR “finisher pig*” OR “finisher hog*” OR porcine NOT "guinea pig*") AND (influenza OR IAV OR flu OR SIV OR “H3N2” OR “H1N1” OR “H1N2” OR “H3N1” OR “H2N3”)) |

†Search strategy and search strings were developed and formatted for selected bibliometric platforms with support from University of Guelph librarians with expertise and experience in systematic review methods.
