# Supplementary material for: Influenza A virus vaccine research conducted in swine from 1990 to May 2018: A scoping review
Source: PLoS One. 2020 Jul 16;15(7):e0236062. doi: 10.1371/journal.pone.0236062 (PMC7365442; doi:10.1371/journal.pone.0236062)
Supplement: S2 Table — Questions and definitions were pre-tested and refined using a minimum sampling of 100 citations and any conflicts or question were resolved before proceeding with screening of all citations. Forms implemented in Distiller-SR. Citations were forwarded to the next level of screening if reviewed as relevant through sequential asking of all questions on a form, or if relevance was determined as unclear by both reviewers. A third reviewer from the team decided on cases of unresolved reviewer disagreement. See S1 Text for explanatory notes and S5 Table for definitions. (DOCX) [file pone.0236062.s005.docx]

**S2 Table. Level 1 (L1) Relevance Screening Form for Title/Abstract Screening.**

Questions and definitions were pre-tested and refined using a minimum sampling of 100 citations and any conflicts or question were resolved before proceeding with screening of all citations. Forms implemented in Distiller-SR.

Citations were forwarded to the next level of screening if reviewed as relevant through sequential asking of all questions on a form, or if relevance was determined as unclear by both reviewers. A third reviewer from the team decided on cases of unresolved reviewer disagreement. See S1 Text for explanatory notes and S5 Table for definitions.

| **Q#** | **Text** | **Answer Text** | **Neutral(1)/ Exclusion(0)** |
| --- | --- | --- | --- |
| 1 | Is this report/study/document about Influenza A virus in/from swine (IAV-S) where swine or direct applicability to swine is the focus? | Yes | 1 |
|  |  | Unclear | 1 |
|  |  | No | 0 |
| 2 | Is the full body text beyond the title/abstract available in English? | Yes | 1 |
|  |  | Unclear | 1 |
|  |  | No | 0 |
| 3 | Is this citation primary research? | Yes | 1 |
|  |  | Unclear | 1 |
|  |  | No, it is a review | 1 |
|  |  | No, it is a white paper, working report, policy paper, issue paper, or guidelines | 0 |
|  |  | No, it is an editorial or commentary | 0 |
|  |  | No, it is another type of publication | 0 |
| 4 | Is the unit of study exclusively at the sub-animal level (e.g. tissue, cellular, molecular, etc. level)? | Yes | 0 |
|  |  | No | 1 |
|  |  | Unclear | 1 |
| 5 | What is the review type as indicated by the authors in the title/abstract? | Narrative or traditional review | 0 |
|  |  | Systematic review without a meta-analysis | 0 |
|  |  | Systematic review and meta-analysis | 0 |
|  |  | Meta-analysis only | 0 |
