# Supplementary material for: Influenza A virus vaccine research conducted in swine from 1990 to May 2018: A scoping review
Source: PLoS One. 2020 Jul 16;15(7):e0236062. doi: 10.1371/journal.pone.0236062 (PMC7365442; doi:10.1371/journal.pone.0236062)
Supplement: S4 Table — Pre-testing and refinement of the Level 3 data charting form was done using full text journals articles (n = 21) until charting was consistent between reviewers. Forms implemented in Distiller-SR. Level 3 includes three modified relevance screening questions carried over from Level 1 and Level 2 forms. See S1 Text for explanatory notes and S5 Table for definitions. (DOCX) [file pone.0236062.s007.docx]

**S4 Table. Level 3 (L3) Relevance Screening and Data Charting Form for Full Text screening.**

Pre-testing and refinement of the Level 3 data charting form was done using full text journals articles (n=21) until charting was consistent between reviewers.

Forms implemented in Distiller-SR. Level 3 includes three modified relevance screening questions carried over from Level 1 and Level 2 forms. See S1 Text for explanatory notes and S5 Table for definitions.

|  |  |  | **Inclusion (1) Exclusion (0)** | |
| --- | --- | --- | --- | --- |
| **Q#** | **Relevance Screening Questions**  **( repeated from L1 & L2)** | **Answer Text (Screening / Characterization options)** |  |  |
| 1 | What is the document type? | Primary research - Conference Proceeding/Abstract | 1 | |
|  |  | Primary research - Academic Journal article | 1 | |
|  |  | Primary research - Thesis/ dissertation | 1 | |
|  |  | Primary research - other | 1 | |
|  |  | Other - not primary research | 0 | |
|  |  | Unclear | 1 | |
|  |  | Not primary research - editorial or commentary | 0 | |
|  |  | A review - narrative | 0 | |
|  |  | A review - systematic with or without MA | 0 | |
|  |  | White paper, guidelines, policy report, etc. | 0 | |
| 2 | Does this research involve vaccine evaluation or development trials in swine? | Yes | 1 | |
|  |  | No | 0 | |
|  |  | Unclear | 1 | |
| 3 | What is the study setting? Select all that apply. (this question was omitted from analysis as it was redundant with relevance question 2 and charting question 2) | in silico |  | |
|  |  | in pig |  | |
| 4 | What is the unit of study/ test population or level of study? Select all that apply. | Individual pig level | 1 | |
|  |  | Groups/ pens/ rooms/ batches of pigs on a site or farm operational level | 1 | |
|  |  | Unclear | 1 | |
|  |  | Exclusively at the sub-pig level | 0 | |
|  |  | Exclusively in a non-pig species | 0 | |
| **Data Charting Questions.** | |  |  | |
| 1 | What is the consideration of vaccines in this study?  Select all that apply. | ONLY IAV-S vaccine development or evaluation |  | |
|  |  | IAV-S vaccine AND other non-vaccine risk factor(s)/intervention(s) |  | |
|  |  | Unclear |  | |
| 2 | What is the study design approach?  Select all that apply. | Descriptive study (e.g. case series) |  | |
|  |  | Hypothesis testing (experimental) |  | |
|  |  | Hypothesis testing (observational) |  | |
|  |  | Hypothesis testing (computer simulation) |  | |
|  |  | Unclear |  | |
|  |  | Other |  | |
| 3 | Is the study within the context of PRDC (porcine respiratory disease complex)? | Yes |  | |
|  |  | No |  | |
|  |  | Unclear |  | |
| 4 | What is the type of IAV-S vaccine study?  Select all that apply. | Challenge studies in swine |  | |
|  |  | Natural exposure studies in swine |  | |
|  |  | No influenza virus exposure |  | |
|  |  | Unclear |  | |
| 5 | What is the vaccine type?  Select all that apply. | Commercial vaccine - inactivated (killed) virus |  | |
|  |  | Commercially produced autogenous vaccine - killed |  | |
|  |  | Commercial vaccine - modified live |  | |
|  |  | Commercial other (sub-unit, particle, DNA) |  | |
|  |  | Experimental vaccine - killed |  | |
|  |  | Experimental vaccine - live |  | |
|  |  | Experimental vaccine other (non-IAV backbone, sub-unit, particle, DNA) |  | |
|  |  | Unclear or not stated |  | |
| 6 | What is the production stage of the study population vaccinated?  Select all that apply. | Gilts (specific to gilt development) |  |  |
|  |  | Sows/gilts (as part of the breeding herd program) |  | |
|  |  | Boars |  | |
|  |  | Neonatal (suckling) pigs |  | |
|  |  | Weaned (nursery) pigs |  | |
|  |  | Grower/finisher pigs |  | |
|  |  | Other |  | |
|  |  | Unclear |  | |
| 7 | What type outcome measures are reported?  Select all that apply. | Production parameters - mortality, FC, ADG |  | |
|  |  | Production parameters - reproduction |  | |
|  |  | Transmissibility (transfer of virus from one pig to another) |  | |
|  |  | Immunologic, pathologic, or pathophysiologic responses of the host |  | |
|  |  | Clinical signs (fever, cough, dyspnea, nasal discharge) |  | |
|  |  | Virus detection (detection of shedding, or of viremia (e.g. PCR, IHC)) |  | |
|  |  | Virus characterization (sequencing or antigenic sub-typing) |  | |
|  |  | Unclear or other |  | |
| 8 | What is the primary author affiliation?  Select all that apply. | University |  | |
|  |  | Pork Production Company (commercial producer) |  | |
|  |  | Independent Research Consultant or Professional |  | |
|  |  | Allied industry (i.e. pharmaceutical, breeding stock, industry association, etc.) |  | |
|  |  | National/sub-national government organizations or government sponsored Funding Agencies |  | |
|  |  | International governmental organization |  | |
|  |  | Non-governmental organizations |  | |
|  |  | Professional organizations |  | |
|  |  | Other |  | |
|  |  | Unclear/Not stated |  | |
| 9 | What region/country is the primary author affiliated with?  Select all that apply. | European Union - 28 |  | |
|  |  | United States |  | |
|  |  | China |  | |
|  |  | Viet Nam |  | |
|  |  | South Korea |  | |
|  |  | Philippines |  | |
|  |  | Other Asian countries |  | |
|  |  | Russian Federation |  | |
|  |  | Other European countries |  | |
|  |  | Mexico |  | |
|  |  | Canada |  | |
|  |  | Brazil |  | |
|  |  | Other Central/South American countries |  | |
|  |  | Oceania |  | |
|  |  | Africa |  | |
|  |  | Unclear/Not stated |  | |
| 10 | What is the funding source for the document? Select all that apply. | University(s) |  | |
|  |  | Pork production company |  | |
|  |  | Allied industry and industry associations (e.g. pharmaceutical, breeding stock, feed company, etc.) | |  |
|  |  | National or sub-national government including government sponsored Funding Agencies |  | |
|  |  | International governmental organizations |  | |
|  |  | Non-governmental organizations |  | |
|  |  | Professional organizations |  | |
|  |  | Other |  | |
|  |  | Unclear/ Not stated |  | |
