# Supplementary material for: Influenza A virus vaccine research conducted in swine from 1990 to May 2018: A scoping review
Source: PLoS One. 2020 Jul 16;15(7):e0236062. doi: 10.1371/journal.pone.0236062 (PMC7365442; doi:10.1371/journal.pone.0236062)
Supplement: S5 Table — (DOCX) [file pone.0236062.s008.docx]

**S5 Table. Definitions Applied in Forms 1, 2 & 3 (L1, L2, L3) for Relevance Screening and Data Charting.**

| **Relevance screening Section of L3 Form:**  **1. What is the document type?**   \| Primary research - Conference Proceeding/Abstract \| New research findings as presented at science based conferences \| \| --- \| --- \| \| Primary research - Academic Journal article \| New findings as published in academic journal literature. \| \| Primary research - Thesis/ dissertation \| New research findings as presented in accredited post-secondary educational programs graduate theses or dissertation requirements. \| \| Primary research - other \| New research findings published as a white paper, working paper, issue paper, policy report, outside of traditional peer reviewed channels for presenting primary research. \| \| Other - not primary research \| Fits no other category \| \| Unclear \| Citation not fitting with the above listed documents types categories. \| \| Not primary research - editorial or commentary \| Often published in peer reviewed literature and authored by those tacitly or explicitly recognized as an authoritative voice on the subject area. Page length is limited and topic coverage is brief opinion or instruction \| \| A review - narrative \| An overview of past facts and findings for the purpose of summating and reflecting on available information on a given subject area but not identification by author as a systematic review. \| \| A review - systematic with or without MA \| As identified by the author in the title/abstract or body of the report. \| \| Report (White paper, working report, policy paper, NGO reports, Association reports, issue papers, guidelines) \| Published documents which may review subject area literature but secondarily as a supportive discourse for purposeful statements or endorsements of an organization’s unified position, agenda, focus of action, or as a contextual statement for recommended guidelines for operation in a subject area.  Report usually of substantial structure and may contain an executive summary (i.e. policy or position reports) and often published by the authoring organization. \|   **2. Does this study involve vaccine evaluation or development trials in swine?**   \| Yes \|  \| \| --- \| --- \| \| no \|  \| \| unclear \|  \|   **3. What is the study setting?**   \| *in silico* \| Research in which computer-based modeling and analysis tools are developed and utilized to predict and elucidate dynamics of biological systems, their design and control, and their evolution. Can include mathematical models or statistical (epidemiologic models). Included modeling citations, unless it was a review document or a commentary, as a primary research paper based on the assumption each run of a model, where even a single parameter is changed, would presenting ‘new findings’, albeit theoretical.  Examples:  Simulation modeling, Agent based , SIR models (Susceptible Infected Recovered), Big data and ‘omic’ analytics such as predictive analytics, phylogenetic , and phylodynamic analyses  Mathematical or computer simulation models refer to dynamic disease transmission models where force of infection varies with changes in the prevalence of infectious and susceptible individuals in a population over time.(Dorjee et al. 2013)  Statistical models explore associations between outcome and explanatory variables and include issues such as statistical characterization of numerical or categorical data, estimating the probalistic future behaviour of a system based on past behavior, extrapolation or interpolation of data based on some best-fit, error estimates of observations, or analysis of data or model generated output. https://serc.carleton.edu/introgeo/mathstatmodels/index.html \| \| --- \| --- \| \| *in pig* \| Tests performed within the whole living organism(pig) as opposed to a tissue extract or dead organism \|   **4. What is the unit of study/ test population?**   \| Individual pig level \| Housing conditions dictate if cluster or individual approach is appropriate for analysis. Not differentiated beyond whole animal level in this scoping review. \| \| --- \| --- \| \| Groups/ pens/ rooms/ or batches of animals/production site/ farm operation level \| \| Exclusively at a sub-pig level \| In vitro, ex vivo (Citation excluded from data charting) \| \| Exclusively in non-pig species \| E.g. studies exclusively of IAV-S in ferrets, humans, dogs, or other non-swine species. (Citation excluded from data charting) \| \| Unclear \| Insufficient details provided to discern unit of study. \|   **Data Charting Section of L3 Form**: Multiple answer options can be selected for each question. All that apply are to be selected for each relevant publication.  **1. What is the consideration of vaccines in this study?**   \| ONLY IAV-S vaccine development or evaluation \| Exclusively IAV vaccine development or evaluation studies and no other IAV-S risk factors or interventions are included in the study design or analysis. \| \| --- \| --- \| \| Intervention/risk factor focus is inclusive of IAV vaccine development/evaluation **AND** one or more other interventions/risk factors. \| On farm disease management programs are most often a combination of adoption of multiple actions. In these studies, observations collected on outcome measures/dependent variables with specific reference to both IAV vaccines and one or more other IAV-S interventions/risk factors as independent or confounding variables (see definitions below) \| \| Unclear \| Insufficient details are provided to confirm if other interventions are included in the study design \| \| **A note on Risk factors and Interventions**:  Definition of intervention as adopted from Oxford English Dictionary by FAO/WHO working group on Salmonella control: “as any action of intervening, or ‘stepping in’, or interfering in any affair, so as to affect its course or issue”, within the context of controlling disease (Pienaar E.D., n.d.). This category includes also studies to identify factors inferred or associated with increasing (risk factors) or decreasing (protective factors or assets) the occurrence of (IAV-S) infection/clinical disease in swine. Interventions can be directed at risk factors, or the absence of an intervention can be a risk factor.  Examples include – protective factors such as products (e.g. vaccines) or programs (e.g. gilt acclimatization) to reduce the risk or impact of infection. Studies ca be inclusive of risk management and biosecurity actions taken as standalone interventions or in conjunction with one or more interventions as part of a program or “best operating practices” to limit exposure, transmission and spread at the animal level or higher (i.e. within a production site, between production sites, regions).  Types of factors: individual characteristics of the pig (pig level) or those that occur in the environmental and affect groups versus individual pigs (farm/operational/regional level).  **Pig level** (factors within the pig): age, co-morbidity, reproductive status, immune status.  **Production site level** (factors within the production site): on-site population size, ownership type (contract, fully owned), production purpose (commercial, breeding stock), site demographic (farrow to finish, sow farm, boar stud, breeder-weaner, nursery, finisher, nursery-finisher, etc.), site animal flow (i.e. all-in/ all-out by site/room/pen, continuous flow, etc.), replacement breeding stock management strategies (quarantine periods, testing, method of acclimatization, etc.), adoption of on-site biosecurity practices, etc.  **Operational level** (factors of entities outside of the site but directly related to the operations of the farm): operational structure (one-site, multi-site), number of sites in operation, total herd size in operation, herd demographic proportions (breed to slaughter, nursery finishing operation only, breed to wean only, etc.) up-stream (sourcing site) infection status, live animal, deadstock, and feed transportation (dedicated, shared, downtime, washing), proximity to other barns (both within the operation and out site of the operation) , adoption of operational best practices (as industry identified good production practices, HACCP principles), etc.  **Regional level** (factors outside the not directly under the control of the operational entity): pig or farm density at different geographic levels, seasonality, regulatory policies/programs, regional disease prevalence, commodity pricing, etc. \| \|   **2. What is the study design-approach?** (definitions as per (Dohoo, Martin, and Stryhn 2009, Chapter7).   \| Descriptive \| Includes case-reports, case-series reports, and surveys**†** designed solely to describe the nature and distribution of outcome events.  Answer what, who, where and when but **not** why and how  Comparisons are not made between exposed versus non-exposed, or treated versus not-treated, and no inferences about associations can be made.  **†**surveys designed to collect information about both an outcome and a potential exposure of interest are to be categorized as cross-sectional (analytic observational study whose outcome frequency measure is prevalence) \| \| --- \| --- \| \| Hypothesis testing *– designed to make comparisons between subgroups of study subjects based on exposure or outcome status for the purpose of making statistical associations or inferences between exposures of interest and outcomes of interest. Includes a comparison group. \| \| \| Experimental \| - includes laboratory (in-house strictly controlled conditions) and controlled field trials (investigator controls allocation of subjects to study groups but study performed under ‘real-world’ conditions, including randomized controlled trials (RCTs) \| \| Observational \| - includes cross-sectional, cohort, case-control , and hybrid studies  - Disease or condition of interest is naturally occurring and the investigator does not control allocation to interventions or exposures. \| \| Computer Simulation \| Simulation modelling (Dorjee 2012)  Predictive analytics is the building and assessment of a model for making predictions. Can involve use of big data, artificial intelligence (Shmueli, 2013). \| \| Unclear \|  \|   **3. Is the study within the context of PRDC (porcine respiratory disease complex)?**  Recognizing the significance of PRDC in the control and management of IAV-S, studies considering study population comorbidity with other swine respiratory pathogens will be identified using this question, including also, studies where the primary focus may have been other non-IAV-S pathogens (e.g. agents of porcine respiratory disease complex such as PRRS, mycoplasma, PRCV, etc.) if some aspect of IAV-S data collection is reported.  **4 What is the type of IAV-S virus exposure?**   \| IAV Challenge trials in swine \| Vaccine trials in swine - challenge trials (allocation to intervention group and disease exposure are deliberate and under the control of the investigator ('Connor and Sargeant, 2014). These studies would normally only be conducted under highly controlled setting where pathogen (IAV) exposure can be limited and controlled. May be trials conducted also for regulatory purposes (i.e. safety/efficacy). \| \| --- \| --- \| \| IAV natural exposure in swine \| Vaccine trials in swine - natural disease exposure. This would include most observational studies and trials conducted in live swine in research or commercial herds. The difference from challenge trials is that the researcher does NOT deliberately expose the swine to the virus. \| \| No influenza virus exposure \| No viral introduction or exposure (usually in a controlled setting to measure response generated by vaccine exposure only) \|   **5. What is the vaccine type?**   \| Commercial Killed \| Killed or inactivated whole IAV-S virus vaccine licenced for sale. \| \| --- \| --- \| \| Experimental Killed \| Killed or inactivated whole IAV-S virus vaccine in development stages or not yet available for sale \| \| Experimental Other \| Other sub-unit, DNA, recombinant (on a non-IAV viral backbone) vaccines in development and not licensed sale or use in swine herds. \| \| Experimental Live \| Live attenuated IAV-S or an attenuated virus vaccine construct on an IAV-S genetic and structural backbone not licensed for sale or use in commercial farms \| \| Unclear \| Insufficient information provided to determine vaccine type. \| \| Commercial Autogenous \| Killed or inactivated vaccine made from custom viral isolate from target farm. Licensed for use and sale only in target farm. (See 9 CFR 113.113 - Autogenous biologics) \| \| Commercial Other \| Other sub-unit, DNA, recombinant (on a non-IAV viral backbone) vaccines licenced for sale and use in swine herds. \| \| Commercial Live \| Live attenuated IAV-S or an attenuated virus vaccine construct on an IAV-S genetic and structural backbone licensed for sale and use in commercial farms \|   **6. What is the production stage of the study population vaccinated?**   \| Gilts in development programs \| Gilts at the development stages in preparation to enter the breeding herd but not yet part of the breeding herd population. This would include vaccination as part of ta gilt development program for example. \| \| --- \| --- \| \| Breeding Herd Females \| Breeding females, inclusive of gilts post introduction to the general breeding herd population. Includes females in gestation and farrowing stages. \| \| Neonatal piglets \| Piglets still in farrowing and on lactating females. \| \| Weaned pigs \| Piglets weaned from the farrowing rooms into housing for young piglets only. Typically an 8 week growing stage for pigs in their fourth week to approximately 12 weeks. \| \| Grower/Finisher \| Facilities where piglet enter at weaning or post nursery stages and remain until slaughter. \| \| Other \| Mixture of ages not associated with a specific production stage \| \| Unclear \| Insufficient information provided on vaccinated population age or production stge. \|   **7. What type outcome metrics are reported?**   \| Immunologic, pathologic, or pathophysiologic responses of the host \| Pathophysiology and immunology - within context of host immunologic response to vaccination and viral infection - physiologic changes or processes within individual swine resulting from IAV-S vaccination humoral, cellular , and tissue architecture responses (ie. lung lesion scores) and also including molecular determinants (such as protein expression) of host immunologic responses, to the vaccine, and viral infection  - inclusive of studies on virus-host interaction- clinical and physiologic changes or processes within individual swine resulting from IAV-S vaccination with or without IAV-S exposure \| \| --- \| --- \| \| Transmissibility \| Measures of viral transfer from one pig to infect another.  **- Not pig-to-human transmission studies** \| \| Production metrics (grow/mort) \| Grow finish metrics for growth such as average daily gain, feed conversion, mortality, etc. \| \| Production metrics (reproduction) \| Reproductive herd parameters such as born alive, pigs weaned per sow , return to service, etc. \| \| Clinical signs \| Manifestation of infection or vaccination including fever, cough, dyspnea \| \| Virus detection \| Includes virus isolation, IHC, in situ hybridization and or PCR, using for example reverse transcription quantitative real-time PCR (RT-qPCR) assays using primers for the conserved M gene90. \| \| Virus characterization \| The outcome measure virus characterization was distinguished from virus detection if viruses detected or isolated in the study population post vaccination were further characterized by genetic sequencing or antigenic sub-typing (ie. beyond standard diagnostic lab services as provided for purposes of detection). \| \| Other or unclear \| Insufficient information provided to determine outcomes measured \|   **8. What is the primary author affiliation (select all that apply)? Primary author was assumed as first author unless stated otherwise.**   \| Under what affiliation does the primary (first) author list in citation title/abstract? Check all that apply if the author lists multiple affiliations. \| \| \| --- \| --- \| \| University \|  \| \| Pork Production Company (commercial producer) \| e.g. Commercial and/or corporate production companies with affiliated contract growers, vertically integrated production such as:  Global top pork producers: <https://www.wattagnet.com/articles/25011-infographic-worlds-top-10-pig-producers>   - WH Group, China - CP Group, Thailand - Wen’s Food Group, China - Triumph Foods, U.S. - BRF, Brazil - NongHyup Agribusiness, South Korea - Cooperl Arc Atlantique, France, - The Maschhoffs, U.S. - Seaboard Corp., U.S. - Vall Companys Grupo, Spain \| \| Independent Research Consultant or Professional \| e.g. Private practitioners (commonly conference proceedings authored by field practitioners in independent practice) \| \| Allied industry (i.e. pharmaceutical, breeding stock, industry association, etc.) \| e.g. National Pork Board (Swine Health Information Center) and Pork Check-off funds used for research , various breeding stock companies, pharmaceutical company annual research awarding, etc. \| \| National or sub-national Government organizations \| e.g. Canadian Food Inspection Agency, US Department of Agriculture, Ontario Ministry of Agriculture and Food including government funded Funding Agencies (e.g. NIH, NSERC, CIHR) \| \| International governmental organization \| E.g. WHO, FAO, EFSA, OIE, STAR-IDAZ. OFFLU \| \| Non-governmental organizations (NGOs) \| Any non-profit, voluntary group which is organized on a local, national or international level and working independent of external control or affiliated political parties and generally engaged in work for aid or development.  e.g. Veterinarians without borders, Bill and Melinda Gates Foundation, Consultative Group on International Agricultural Research (CGIAR), National Institute for Animal Agriculture (NIAA) \| \| Professional organizations / associations \| American Association of Swine Veterinarians, European Association of Porcine Health Management \| \| Other \|  \| \| Unclear/ Not-stated \|  \|   **9. What country/ region is the primary author affiliated with?(**countries lumped into regions if not a global top 10 pork producer)   \| China \| Does not include Taiwan \| \| --- \| --- \| \| Viet Nam \|  \| \| South Korea \|  \| \| Philippines \|  \| \| Other Asian countries (not in the global top 10 pork producers) \| Not China, Viet Nam, South Korea, Philippines but inclusive of other Asian countries (including Taiwan) not included in the listing of top 10 global pork producers (in 1000MT CWE) -USDA FAS \| \| European Union-28 \| Austria, Belgium, Bulgaria , Croatia, Cyprus , Czech Republic, Denmark , Estonia, Finland , France, Germany, Greece, Hungary, Ireland , Italy , Latvia, Lithuania, Luxembourg, Malta, Netherlands, Poland , Portugal, Romania, Slovakia, Slovenia, Spain , Sweden, United Kingdom \| \| Russian Federation \|  \| \| Other European countries not in the global top 10 pork producers \| Albania, Armenia. Belarus, Gibraltar, Iceland, Kosovo, Liechtenstein, Macedonia, Norway, Switzerland, Turkey, Ukraine, and Vatican City State \| \| United States \|  \| \| Mexico \|  \| \| Canada \|  \| \| Brazil \|  \| \| Other Caribbean, Central/South American countries not in the global top 10 pork producers \| Central America: Belize, Costa Rica, El Salvador, Guatemala, Honduras, Nicaragua, Panama  South America (**not including Brazil**): Venezuela, Argentina, Uruguay, Bolivia, Suriname, Peru, Chile, Paraguay, Colombia, Guyana, Ecuador, French Guiana, Falkland Islands.  Caribbean: see <https://en.wikipedia.org/wiki/List_of_Caribbean_countries_by_population> \| \| Oceania \| Australia, New Zealand, Melanesia, Micronesia, Polynesia \| \| Africa \| See: <https://www.countries-ofthe-world.com/countries-of-africa.html> \|   **10. What is the funding source for the document (select all that apply)?**   \| University(s) \| See definitions above for Question 12. Author affiliations \| \| --- \| --- \| \| Pork Production Company (commercial producer) \| \| Independent Research Consultant or Professional \| \| Allied industry and industry associations \| \| National or sub-national government organizations \| \| International governmental organization \| \| Non-governmental organizations \| \| professional organizations \| \| Other \| \| Unclear/Not-stated \| |
| --- | --- | --- | --- | --- | --- | --- | --- | --- | --- | --- | --- | --- | --- | --- | --- | --- | --- | --- | --- | --- | --- | --- | --- | --- | --- | --- | --- | --- | --- | --- | --- | --- | --- | --- | --- | --- | --- | --- | --- | --- | --- | --- | --- | --- | --- | --- | --- | --- | --- | --- | --- | --- | --- | --- | --- | --- | --- | --- | --- | --- | --- | --- | --- | --- | --- | --- | --- | --- | --- | --- | --- | --- | --- | --- | --- | --- | --- | --- | --- | --- | --- | --- | --- | --- | --- | --- | --- | --- | --- | --- | --- | --- | --- | --- | --- | --- | --- | --- | --- | --- | --- | --- | --- | --- | --- | --- | --- | --- | --- | --- | --- | --- | --- | --- | --- | --- | --- | --- | --- | --- | --- | --- | --- | --- | --- | --- | --- | --- | --- | --- | --- | --- | --- | --- | --- | --- | --- | --- | --- | --- | --- | --- | --- | --- | --- | --- | --- | --- | --- | --- | --- | --- | --- | --- | --- | --- | --- | --- | --- | --- | --- | --- | --- | --- | --- | --- | --- | --- | --- | --- | --- | --- | --- | --- |
