# Supplementary material for: Influenza A virus vaccine research conducted in swine from 1990 to May 2018: A scoping review
Source: PLoS One. 2020 Jul 16;15(7):e0236062. doi: 10.1371/journal.pone.0236062 (PMC7365442; doi:10.1371/journal.pone.0236062)
Supplement: S6 Table — (DOCX) [file pone.0236062.s009.docx]

**S6 Table. Summary of Journal Article Counts by Journal Titles and Listing Status as Open Access (OA) on Directory of Open Access Journals (DOAJ)** <https://doaj.org>

| **Journal Title** | **Count** | **Cumm. Count** | **Cumm. %** | **DOAJ - no** | **DOAJ - yes** | **No. Articles DOAJ** |
| --- | --- | --- | --- | --- | --- | --- |
| Vaccine | 36 | 36 | 21% | 1 |  |  |
| Veterinary Microbiology | 14 | 50 | 29% | 1 |  |  |
| PLoS ONE | 11 | 61 | 36% |  | 1 | 11 |
| Veterinary Immunology and Immunopathology | 8 | 69 | 41% | 1 |  |  |
| Journal of Virology | 7 | 76 | 45% | 1 |  |  |
| Virus Research | 6 | 82 | 48% | 1 |  |  |
| Influenza and other Respiratory Viruses | 6 | 88 | 52% |  | 1 | 6 |
| Viral Immunology | 5 | 93 | 55% | 1 |  |  |
| Veterinary Research | 5 | 98 | 58% | 1 |  |  |
| Journal of General Virology | 5 | 103 | 61% | 1 |  |  |
| Clinical and Vaccine Immunology | 5 | 108 | 64% | 1 |  |  |
| Canadian Journal of Veterinary Research | 4 | 112 | 66% | 1 |  |  |
| Virology | 3 | 115 | 68% | 1 |  |  |
| Transboundary and Emerging Diseases | 3 | 118 | 69% | 1 |  |  |
| Scientific Reports | 3 | 121 | 71% |  | 1 | 3 |
| Journal of Immunology | 3 | 124 | 73% | 1 |  |  |
| Frontiers in Immunology | 3 | 127 | 75% |  | 1 | 3 |
| Veterinary Record | 2 | 129 | 76% | 1 |  |  |
| PLoS Pathogens | 2 | 131 | 77% |  | 1 | 2 |
| PLoS currents | 2 | 133 | 78% |  | 1 | 2 |
| Journal of Swine Health and Production | 2 | 135 | 79% | 1 |  |  |
| Journal of Controlled Release | 2 | 137 | 81% | 1 |  |  |
| International Journal of Pharmaceutics | 2 | 139 | 82% | 1 |  |  |
| Avian Diseases | 2 | 141 | 83% | 1 |  |  |
| Archives of Virology | 2 | 143 | 84% | 1 |  |  |
| American Journal of Veterinary Research | 2 | 145 | 85% | 1 |  |  |
| Zoonoses and Public Health | 1 | 146 | 86% | 1 |  |  |
| Veterinary Pathology | 1 | 147 | 86% | 1 |  |  |
| Veterinární Medicína | 1 | 148 | 87% |  | 1 | 1 |
| The Journal of Microbiology | 1 | 149 | 88% | 1 |  |  |
| Science Translational Medicine | 1 | 150 | 88% | 1 |  |  |
| Research in Veterinary Science | 1 | 151 | 89% | 1 |  |  |
| Preventive Veterinary Medicine | 1 | 152 | 89% | 1 |  |  |
| Npj Vaccines | 1 | 153 | 90% |  | 1 | 1 |
| Journal of Veterinary Diagnostic Investigation | 1 | 154 | 91% | 1 |  |  |
| Journal of Surgical Research | 1 | 155 | 91% | 1 |  |  |
| Journal of Investigative Dermatology | 1 | 156 | 92% | 1 |  |  |
| Journal of Infectious Diseases | 1 | 157 | 92% | 1 |  |  |
| Journal of Functional Foods | 1 | 158 | 93% | 1 |  |  |
| Journal of Animal Science | 1 | 159 | 94% | 1 |  |  |
| Japanese Journal of Veterinary Research | 1 | 160 | 94% | 1 |  |  |
| Immunology and Infectious Diseases | 1 | 161 | 95% | 1 |  |  |
| European Journal of Immunology | 1 | 162 | 95% | 1 |  |  |
| Emergence and Control of Zoonotic Ortho- and Paramyxovirus Diseases | 1 | 163 | 96% | 1 |  |  |
| Comparative Immunology, Microbiology and Infectious Diseases | 1 | 164 | 96% | 1 |  |  |
| Cellular Immunology | 1 | 165 | 97% | 1 |  |  |
| BMC Veterinary Research | 1 | 166 | 98% |  | 1 | 1 |
| American Journal of Clinical Nutrition | 1 | 167 | 98% | 1 |  |  |
| Agricultural Systems | 1 | 168 | 99% | 1 |  |  |
| Agricultural Sciences in China | 1 | 169 | 99% | 1 |  |  |
| Acta Veterinaria (Beograd) | 1 | 170 | 100% |  | 1 | 1 |
| **Total** |  |  |  | 41 | 10 | 31 |

Cumm. = cumulative
