# Supplementary material for: Influenza A virus vaccine research conducted in swine from 1990 to May 2018: A scoping review
Source: PLoS One. 2020 Jul 16;15(7):e0236062. doi: 10.1371/journal.pone.0236062 (PMC7365442; doi:10.1371/journal.pone.0236062)
Supplement: S7 Table — †There was no duplicative indexing of proceedings by CABD and WOS; the AASV Library citation was deduplicated if indexed also in CABD and/or WOS. (DOCX) [file pone.0236062.s010.docx]

**S7 Table. Conference Proceeding Counts and Conference Event Counts for Conference Series, by Bibliographic Database Source**†.

|  | **AASV Library** | | **CABD** | | **WOS** | | **Overall** | |
| --- | --- | --- | --- | --- | --- | --- | --- | --- |
| **Meeting/Congress/Symposium/ Conference Title** | **No. Cit.** | **No. Events** | **No. Cit.** | **No. Events** | **No. Cit.** | **No. Events** | **No. Cit.** | **Total Events** |
| Annual Meeting of the American Association of Swine Veterinarians | 49 | 13 | 11 | 2 | 15 | 5 | **75** | 20 |
| International Pig Veterinary Society Congress | 48 | 9 |  |  |  |  | **48** | 9 |
| Allen D. Leman Swine Conference | 25 | 12 |  |  |  |  | **25** | 12 |
| International Symposium on Emerging and Re-emerging Pig Diseases |  |  | 6 | 1 |  |  | **6** | 1 |
| Joint Meeting ESPHM /PVSGB‡ |  |  | 4 | 4 |  |  | **4** | 4 |
| Annual Meeting of the American Association of Immunologists |  |  |  |  | 4 | 3 | **4** | 3 |
| ISU Swine Disease Conference for Swine Practitioners | 3 | 3 |  |  |  |  | **3** | 3 |
| Asian Pig Veterinary Society Congress |  |  | 3 | 1 |  |  | **3** | 1 |
| Meeting of the United States Animal Health Association |  |  | 2 | 2 |  |  | **2** | 2 |
| International Symposium on Neglected Influenza Viruses |  |  | 1 | 1 |  |  | **1** | 1 |
| International Congress of Immunology |  |  |  |  | 1 | 1 | **1** | 1 |
| Experimental Biology |  |  |  |  | 1 | 1 | **1** | 1 |
| Biophotonics and Immune Responses |  |  |  |  | 1 | 1 | **1** | 1 |
| International Conference on Production Disease in Farm Animals |  |  | 1 | 1 |  |  | **1** | 1 |
| Total | **125** | **37** | **28** | **12** | **22** | **11** | **175** | 60 |

AASV Library = AASV Swine Information Library Online; CABD = Cab Direct; WOS = Web of Science; Cit. = proceeding citation;

†There was no duplicative indexing of proceedings by CABD and WOS; the AASV Library citation was deduplicated if indexed also in CABD and WOS; ‡Joint Meeting European Symposium of Porcine Health Management / Meeting of the Pig Veterinary Society of Great Britain.
